# Structure function analysis of ADP-dependent cyanobacterial phosphofructokinase reveals new phylogenetic grouping in the PFK-A family

**DOI:** 10.1016/j.jbc.2024.107868

**Published:** 2024-10-10

**Authors:** Lu Shen, Carmen Peraglie, David Podlesainski, Christina Stracke, Ravi Shankar Ojha, Frauke Caliebe, Markus Kaiser, Karl Forchhammer, Martin Hagemann, Kirstin Gutekunst, Jacky L. Snoep, Christopher Bräsen, Bettina Siebers

**Affiliations:** 1 Molecular Enzyme Technology and Biochemistry (MEB), Environmental Microbiology and Biotechnology (EMB), Centre for Water and Environmental Research (CWE), Faculty of Chemistry, University of Duisburg-Essen, Essen, Germany; 2 Chemical Biology, Centre of Medical Biotechnology (ZMB), Faculty of Biology, University of Duisburg-Essen, Essen, Germany; 3 Molekulare Pflanzenphysiologie, University of Kassel, Kassel, Germany; 4 Microbiology, University of Tübingen, Tübingen, Germany; 5 Plant Physiology, University of Rostock, Rostock, Germany; 6 Biochemistry, University of Stellenbosch, Stellenbosch, South Africa; 7 Molecular Cell Biology, Vrije Universiteit Amsterdam, Amsterdam, The Netherlands

**Keywords:** PFK-A superfamily, ADP-dependent PFK-A, allosteric regulation, ATP, ADP, PPi signature binding motifs, cyanobacteria

## Abstract

Depending on the light conditions, photosynthetic organisms switch between carbohydrate synthesis or breakdown, for which the reversibility of carbohydrate metabolism, including glycolysis, is essential. Kinetic regulation of phosphofructokinase (PFK), a key-control point in glycolysis, was studied in the cyanobacterium *Synechocystis* sp. PCC 6803. The two PFK iso-enzymes (PFK- A1, PFK-A2), were found to use ADP instead of ATP, and have similar kinetic characteristics, but differ in their allosteric regulation. PFK-A1 is inhibited by 3-phosphoglycerate, a product of the Calvin-Benson-Bassham cycle, while PFK-A2 is inhibited by ATP, which is provided by photosynthesis. This regulation enables cyanobacteria to switch PFK off in light, and on in darkness. ADP dependence has not been reported before for the PFK-A enzyme family and was thought to be restricted to the PFK-B ribokinase superfamily. Phosphate donor specificity within the PFK-A family could be related to specific binding motifs in ATP-, ADP-, and PPi-dependent PFK-As. Phylogenetic analysis revealed a distribution of ADP-PFK-As in cyanobacteria and in a few alphaproteobacteria, which was confirmed in enzymatic studies.

Cyanobacteria, the only prokaryotes using oxygenic photosynthesis to fuel CO_2_ fixation, are gaining increasing interest as green bio-factories (1). *Synechocystis* sp. PCC 6803 (hereafter named *Synechocystis*) is a cyanobacterial model organism that possesses a broad metabolic versatility (2). During photoautotrophic growth, *Synechocystis* uses the Calvin-Benson-Bassham (CBB) cycle for CO_2_ fixation, leading to the formation of 3-phosphoglycerate (3PG), which can be used in biosynthetic reactions, or stored as glycogen using the anabolic (gluconeogenesis) Embden-Meyerhof-Parnas (EMP) pathway, which has several overlapping reactions with the CBB cycle (3, 4). In the dark heterotrophic lifestyle, glycogen is degraded *via* catabolic pathways, of which four have been reported in *Synechocystis* (Fig. 1): (i) the EMP pathway, (ii) the pentose phosphate (PP) pathway, consisting of the oxidative (OPP) and non-oxidative (NOPP) branches, (iii) the Entner-Doudoroff (ED) pathway (5), and (iv) the phosphoketolase (PK) pathway (6, 7). The PK pathway, however, is inactive under normal diurnal growth conditions (6). The occurrence of the ED pathway remains unclear due to claims that it is incomplete in the majority of cyanobacteria, including *Synechocystis*, owing to the absence of 6-phosphogluconate dehydratase (8). Consequently, this limits *Synechocystis* to the EMP and the OPP pathway for sugar oxidation. In addition to pure auto- or heterotrophic metabolism, *Synechocystis* can also display photomixotrophic growth, using glucose during the light period (1). The regulatory mechanism enabling *Synechocystis* to switch between autotrophic/anabolic and heterotrophic/catabolic metabolic pathways is not well understood. Of the glucose metabolism pathways, only the EMP pathway is reversible, and it must therefore be involved in the regulatory mechanism.

**Figure 1:**
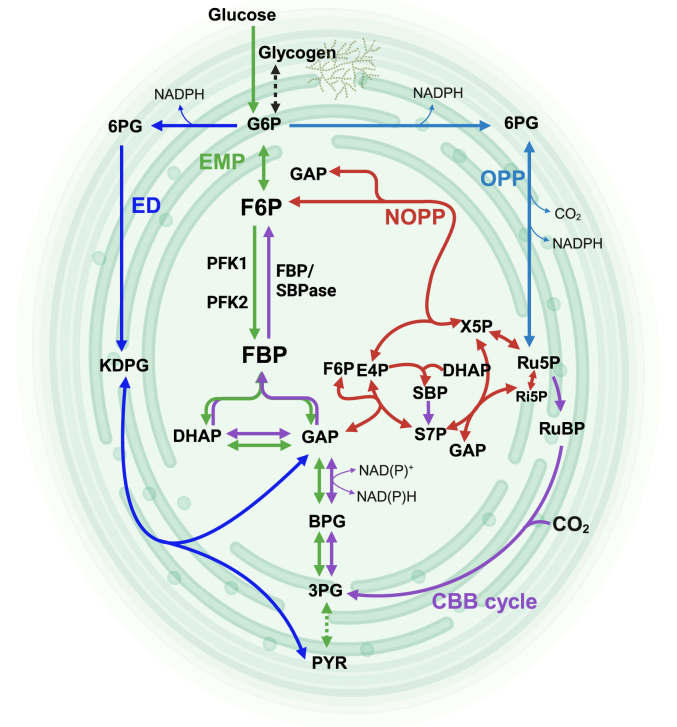
**Overview of pathways involved in CO**_**2**_**-fixation and carbohydrate metabolism in *Synechocystis* sp. PCC6803.** The key enzymes of the reversible EMP pathway, phosphofructokinase (PFK) and the bifunctional fructose-1,6-bisphosphatase/sedoheptulose-1,7-bisphosphatase (FBP/SBPase) are shown. Pathways are differentiated by arrow color: The EMP pathway is shown in *green*, the OPP pathway in *cyan*, the ED pathway (under discussion) in *blue*, the CBB cycle in *purple*, and the NOPP pathway in *red*. The PK pathway was omitted for clarity. 3PG, 3-phosphoglycerate; 6PG, 6-phosphogluconate; BPG, 1,3-bisphosphoglycerate; CBB, Calvin–Benson–Bassham cycle; DHAP, dihydroxyacetone phosphate; E4P, erythrose 4-phosphate; ED, Entner-Doudoroff pathway; EMP, Embden–Meyerhof–Parnas pathway; F6P, fructose 6-phosphate; FBP, fructose 1,6-bisphosphate; GAP, glyceraldehyde 3-phosphate; G6P, glucose 6-phosphate; KDPG, 2-keto-3-deoxy-6-phosphogluconate; OPP, oxidative pentose phosphate pathway; NOPP, non-oxidative pentose phosphate pathway; PYR, pyruvate; Ru5P, ribulose 5-phosphate; RuBP, ribulose 1,5-bisphophate; Ri5P, ribose 5-phosphate; S7P, sedoheptulose 7-phosphate; SBP, sedoheptulose 1,7-bisphosphate; X5P, xylulose 5-phosphate. The figure was created using BioRender.com.

Traditionally, the irreversible kinases, and in particular PFK, are seen as important control points of the glycolytic EMP pathway in Bacteria and Eukaryotes. Depending on the mode of operation, the irreversible phosphofructokinase (PFK) must be active (catabolic mode), or inhibited (anabolic mode), during which the enzyme is bypassed in the gluconeogenetic direction as part of the CBB cycle *via* a fructose 1,6-bisphosphatase (FBPase) reaction (9, 10). So far three different types of PFKs have been described that vary in their respective phosphate donor and regulatory properties: ATP-PFKs, ADP-PFKs, and PP_i_-PFKs. Allosteric ATP-PFKs and PP_i_-PFKs are members of the PFK-A family, with typically phosphoenolpyruvate (PEP) and ATP acting as allosteric regulators in bacteria (11), and a more complex structure and more extensive regulation in eukaryotes (12). Non-allosteric ATP-PFKs and ADP-PFKs are members of the PFK-B family within the ribokinase superfamily (13). PP_i_-dependent enzymes have been reported for Archaea, *i.e. Thermoproteus tenax*, Bacteria, as well as Eukaryotes, including protists, fungi and plants (14, 15, 16, 17). ADP-dependent phosphorylation of fructose 6-phosphate (F6P) has so far only been reported in Archaea (PFK-Bs), *i.e.* hyperthermophilic Euryarchaeota such as *Thermococcus zilligii*, *Pyrococcus furiosus* and *Archaeoglobus fulgidus*, for a review see (18, 19, 20, 21).

Based on kinetic analyses in cell extracts (4), the two PFK isoenzymes expressed in *Synechocystis* (PFK-A1, *sll1196*; PFK-A2, *sll0745*), were classified as non-allosteric ATP-PFKs from the PFK-B family, although sequence comparisons indicated them to be members of the PFK-A family (22). To resolve this inconsistency we cloned, expressed, purified, and characterized the two *Synechocystis* PFK isoenzymes. Both isoenzymes used exclusively ADP as cosubstrate, and were allosterically regulated by 3PG (PFK-A1) or ATP (PFK-A2), and displayed a typical PFK-A fold. While the fructose 6-phosphate (F6P) binding site is conserved in the PFK-A family, structural differences and specific motifs for co-substrate binding, depending on the phosphate donor, were identified. The phylogenetic analysis demonstrates that ADP-PFK-As form a monophyletic group of ”short” 40 kDa PFK-As, reflecting their common ancestry, alongside ATP-PFK-As and PP_i_-dependent PFK-As.

## Results

### Expression and purification of *Synechocystis* PFK isoenzymes

To characterize the two PFK isoenzymes from *Synechocystis*, the encoding genes (PFK-A1, *sll1196*; PFK-A2, *sll0745*) were cloned using the pET expression system with N-terminal His-tag, and purified *via* immobilized metal ion affinity chromatography (IMAC) and size exclusion chromatography (SEC) (Fig. S1).

The molecular mass of PFK-A1 and PFK-A2 under denaturing conditions (SDS-PAGE) was approximately 39 kDa and 42 kDa, respectively, being in good agreement with the calculated molecular mass of 40.47 kDa for PFK-A1 and 43.64 kDa for PFK-A2. The native molecular mass was first determined by SEC and yielded 80 kDa for both, PFK- A1 and PFK-A2 indicating a homodimeric structure for both enzymes (Fig. S2). Native mass spectrometry confirmed a predominantly dimeric structure of PFK-A2 but also identified a tetrameric form of this protein occurring to a lesser extent (Fig. S3). In contrast, MS revealed a predominantly tetrameric structure of PFK-A1 with only traces of the dimeric form present in the given buffer conditions (Fig. S4*A*). Furthermore, F6P had a positive impact on PFK-A1 stability in the MS-compatible buffer ammonium acetate, resulting in much improved MS signal intensities (Fig. S4*B*).

### Enzyme characterization of *Synechocystis* PFK isoenzymes

**Figure 2:**
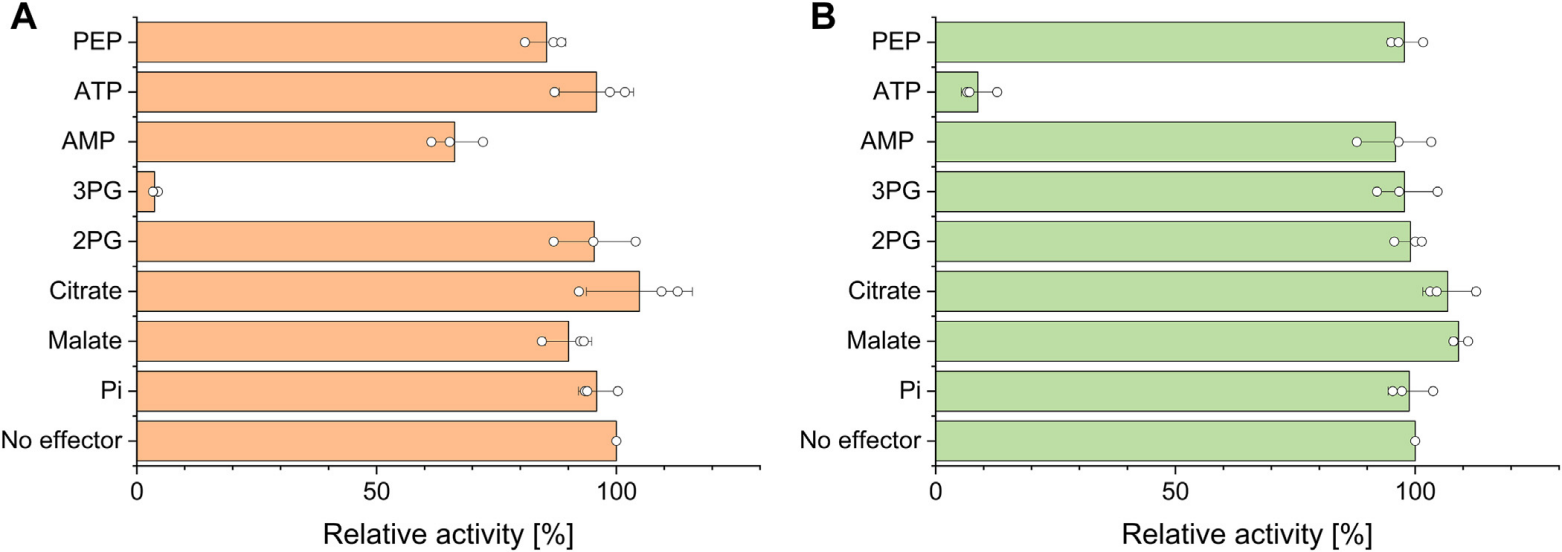
**Effector studies for PFK-A isoenzymes from *Synechocystis* sp. PCC 6803. The influence of different metabolites/effectors on ADP-PFK-A1 (A) and ADP_PFK-A2 is shown.** The influence of different metabolites/effectors on ADP-PFK-A1 (*A*) and ADP-PFK-A2 (*B*) is shown. Assays were performed with subsaturating concentrations of F6P (0.08 mM for PFK-A1 and 0.07 mM for PFK-A2) and ADP (0.4 mM for PFK-A1 and 0.2 mM for PFK-A2). The different effectors were tested at a concentration of 1 mM. The relative activity (%) in comparison to the control without effector (100%) are shown. The specific activity without effector for ADP-PFK-A1 was 1.35 U/mg and for ADP-PFK-A2 2.77 U/mg. The means and standard deviations for three technical replicates (n = 3) are shown.

In contrast to previous reports on ATP-PFK activity in crude extracts (4), we observed no activity of the two purified PFKs with ATP (or PP_i_) as phosphate donors, and both enzymes were completely dependent on ADP for catalytic activity (see Fig. S5). Strong inhibition of PFK-A1 by 3PG (4% residual activity, at 1 mM 3PG) was observed, while the classic effectors ATP and PEP (96% and 86% residual activity, at 1 mM concentrations) did not affect the activity much (see Fig. 2*A*). For PFK-A2, a strong inhibition by ATP (9% residual activity, at 1 mM) was observed, while none of the other tested carbon intermediates affected the enzyme activity significantly (see Fig. 2*B*).

**Figure 3:**
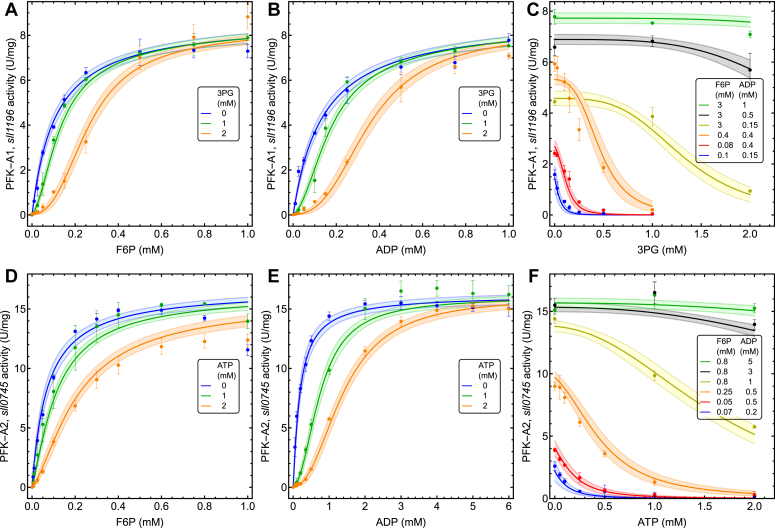
**Characterization of the *Synechocystis* PFK-A isoenzymes.** (*A*-*C*) PFK-A1, (*sll1196*) kinetics and the effect of 3PG. *A*, F6P saturation, with 3 mM ADP, and three different 3PG concentrations, (*B*) ADP saturation, with 3 mM F6P, and three different 3PG concentrations, (*C*) the effect of 3PG, with six different combinations of F6P and ADP concentrations; (*D*–*F*) PFK-A2, (*sll0745*) kinetics and the effect of ATP. (*D*) F6P saturation with 3 mM ADP and three different ATP concentrations, (*E*) ADP saturation, with 0.8 mM F6P, and three different ATP concentrations, (*F*) ATP inhibition, with six different combinations of F6P and ADP concentrations. The MWC model (Equation 1) fit is shown with parameter values given in Table 1; colored bands indicate the 95% confidence intervals. Error bars indicate the standard deviation of the mean with n = 3 (technical replicates).

Saturation curves for F6P and ADP were made for PFK-A1 with or without 3PG (Fig. 3, *A* and *B*) and for PFK-A2 with or without ATP (Fig. 3, *D* and *E*), and inhibitor titrations for 3PG or ATP at different F6P and ADP concentrations (Fig. 3, *C* and *F*) are shown for both isoenzymes. Both enzymes shift between a hyperbolic and sigmoidal saturation curve as a function of the allosteric effector, and a Monod-Wyman-Changeux (MWC) model (Equation 1), which is often used for describing PFK kinetics (23, 24), was fitted to the data (parameter values shown in Table 1).

$$\sigma_{1}=\frac{F6P}{K_{F6P}}\;{\;\sigma }_{2}=\frac{\text{ADP}}{K_{\text{ADP}}}{\;\alpha }_{1}=\frac{3\text{PG}}{K_{3\text{PG}}}$$

$$c_{1}=\frac{K_{F6P}}{K_{T_{F6P}}}{\;L}_{0}=\frac{T_{0}}{R_{0}}{\;\alpha }_{2}=\frac{\text{ATP}}{K_{\text{ATP}}}$$

(1) $$\frac{v_{PF{K-A}_{i}}}{V_{M}}=\frac{\sigma_{1}\sigma_{2}{\left(1+\sigma_{1}\right)}^{\left(n-1\right)}{\left(1+\sigma_{2}\right)}^{\left(n-1\right)}}{{\left(1+\sigma_{1}\right)}^{n}{\left(1+\sigma_{2}\right)}^{n}+L_{0}{\left(1+\alpha_{i}\right)}^{n}{\left(1+c_{1}\sigma_{1}\right)}^{n}}$$

with i = 1 or two for PFK-A1 and PFK-A2 respectively.

**Table 1:** Parameter values for PFK-A1 and PFK-A2

Parameter	PFK-A11	PFK-A2	(Units)
*V*_*M*_	9.1 (*±*0.19)	17.6 (*±*0.33)	(U mg^−1^)
*K*F6P	0.11 (*±*0.01)	0.07 (*±*0.01)	(mM)
*K*ADP	0.14 (*±*0.01)	0.17 (*±*0.03)	(mM)
*n*	4.0 (*±*0.39)	2.5 (*±*0.29)	
*K*3PG	0.11 (*±*0.02)		(mM)
*K*ATP		0.20 (*±*0.06)	(mM)
*k*cat	5.9	12.3	(s^−1^)
*L*_0_	135	35.4	
*c*_*1*_	0.01	0.11	

Parameter values for the two enzymes are shown with standard error of the mean in brackets.

The MWC model was able to describe the obtained experimental data accurately, with similar characteristics for both enzymes, and the nature of the allosteric inhibitor, 3PG or ATP, as the most notable difference. For both enzymes the inhibitory effect of 3PG or ATP was diminished with increasing concentrations of F6P and ADP (Fig. 3, *C* and *F*).

### Structural and phylogenetic analyses of PFK enzymes

**Figure 4:**
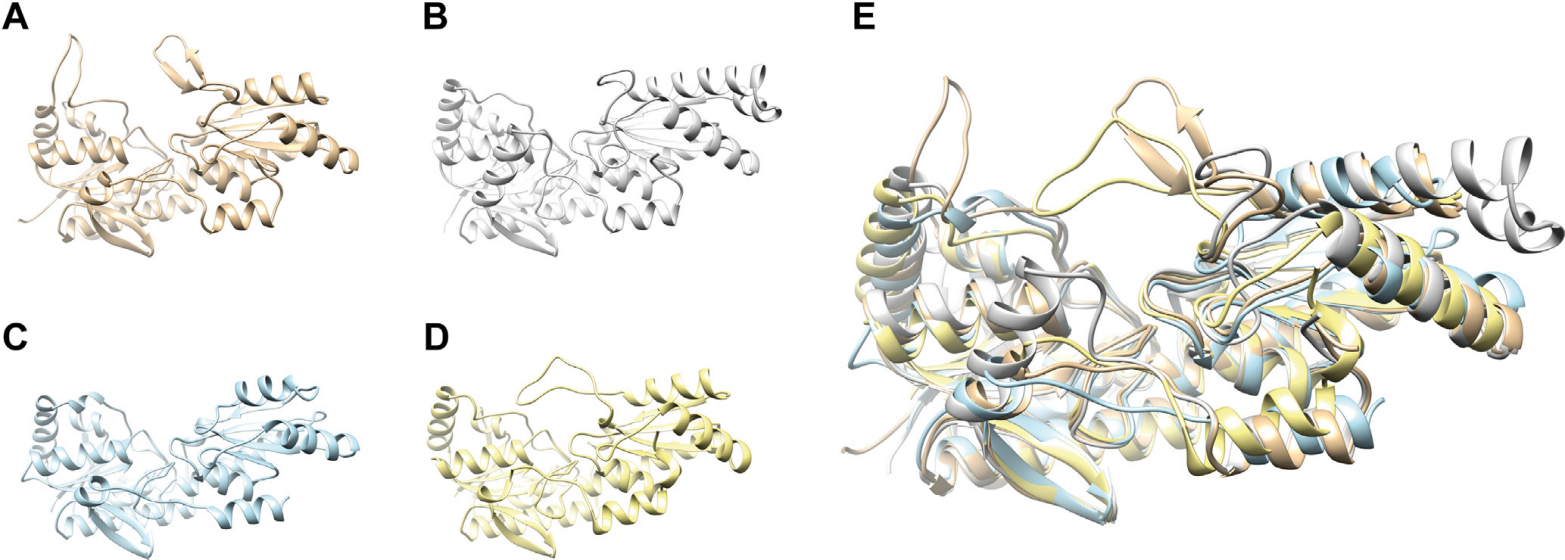
**Structural comparison of the *Synechocystis* PFK-A1 with other PFK-As.** Ribbon representations are shown for the monomers of *Synechocystis* ADP-dependent PFK-A1 (Sll1196, alphafold, *brown*) (*A*) and PFK-A2 (Sll0745, alphafold, *grey*) (*B*) in comparison with the ATP-dependent PFK-A from *S. aureus* (5xz9, crystal structure, *blue*) (30) (*C*) and PP_i_-dependent PFK-A from *T. tenax* (TTX 1277, alphafold, *yellow*) (*D*). The superimposition of all four monomers shown in (*E*) clearly shows that the cyanobacterial ADP-PFK-As adopt the typical PFK-A fold.

Sequence similarity and structural (alphafold) analyses showed that the *Synechocystis* ADP-PFKs belong to the PFK-A family, adopting its characteristic fold (see Fig. 4) rather than the ribokinase fold (PFK-B family) observed in ADP-PFKs primarily found in archaeal species (see Fig. S6 for sequence alignment, and Fig. S7 for a comparison of the PFK-A and PFK-B family folds.

**Figure 5:**
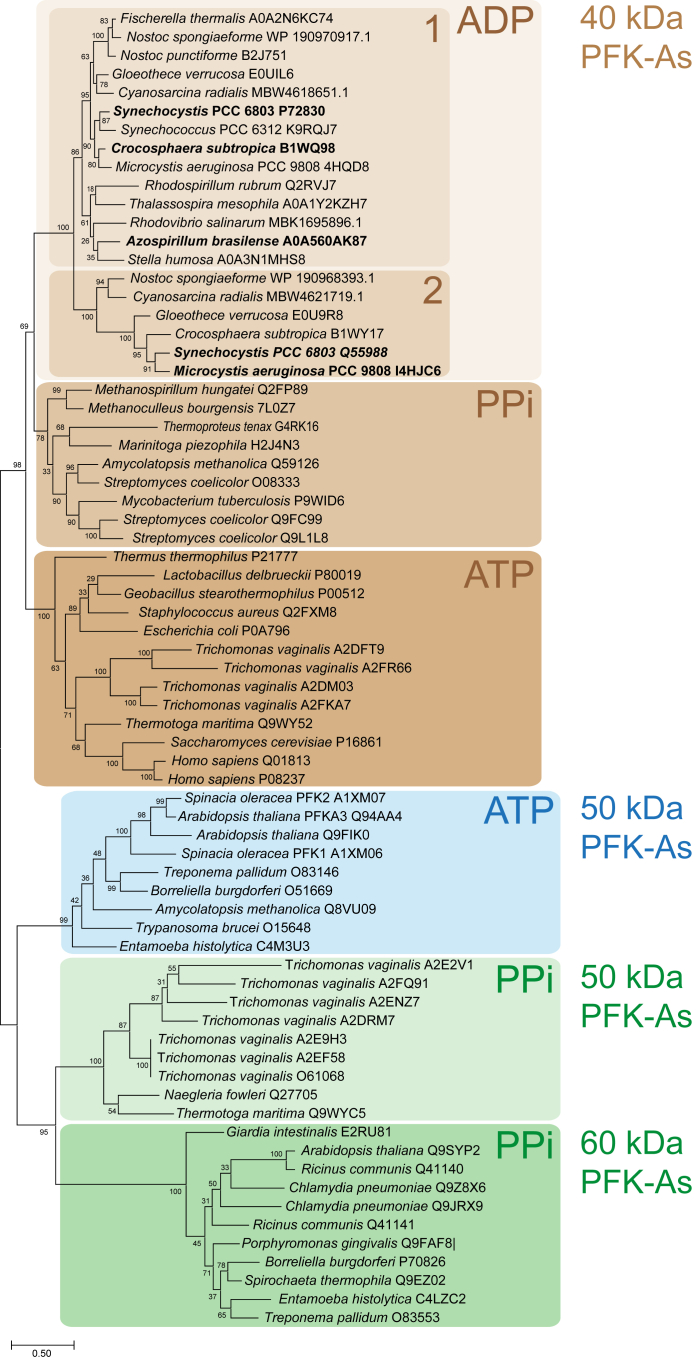
**Phylogenetic analysis of the PFK-A family.** The phylogenetic affiliations of the cyanobacterial and alphaproteobacterial ADP-PFK-As within the PFK-A superfamily are given (with uniprot accession number if available, otherwise Genbank or NCBI accessions). Enzymes characterized in this study are marked in boldface. The “40 kDa subgroup”, include ATP-PFK-As (previously named Group 1 according to (15, 27, 28), dark brown), as well as PP_i_- and ADP-PFK-As (previously designated as Group 3, shown in *brown* and *light brown* respectively). The ADP-PFK-As subcluster is further subdivided into ADP-PFK-A1 (Sll1196) and ADP-PFK-A2 (Sll0745) homologs. The “50 kDa subgroup” (group 2a) of ATP-PFK-As is shown in blue, and the “50 kDa” (group 2 “short”) and the “60 kDa subgroup” (group 2 “long”) of PP_i_-dependent PFK-As are shown in *light and dark green*, respectively. The evolutionary history was inferred by using the Maximum Likelihood method and the Le/Gascuel model (62). The model was selected based on the lowest Bayesian information criterion value using the “Find best protein model” option implemented in the MEGA11 package. The tree with the highest log likelihood (−29534.88) is shown. The percentage of trees in which the associated taxa clustered together is shown next to the branches. Initial tree(s) for the heuristic search were obtained automatically by applying Neighbor-Join and BioNJ algorithms to a matrix of pairwise distances estimated using the JTT model, and then selecting the topology with superior log likelihood value. The tree is drawn to scale, with branch lengths measured in the number of substitutions per site. This analysis involved 71 amino acid sequences. All positions containing gaps and missing data were eliminated (complete deletion option). There was a total of 286 positions in the final dataset. Evolutionary analyses were conducted in MEGA11 (61).

ADP-PFK-A homologues were identified almost exclusively in cyanobacteria and alphaproteobacteria with only rare representatives in other bacterial lineages (mostly from the PVC (Planctomycetota, Verrucomicrobiota, and Chlamydiota) superphylum, FCB (Fibrobacterota, Chlorobiota, and Bacteroidota) superphylum, and the Thermodesulfobacteriota). These ADP-PFK sequences share more than 40% sequence identity whereas ATP- and PP_i_-PFK-As show less than 40% identity and differ in certain sequence features (see below). Phylogenetic analyses reveal a distinct distribution of PFKs based on size and phosphate donor specificity (Fig. 5). In accordance with their determined molecular mass (see above), the ADP-PFK-As form a monophyletic group of “short” 40 kDa PFK-As, along with ATP-PFK-As from bacteria (also including the sequences derived from gene duplications from animals and fungi) and PP_i_-dependent PFK-As from Bacteria and Archaea. The 50 kDa ATP-PFK-As, as well as the 50 kDa and 60 kDa PP_i_-PFK-As from plants, protists, and spirochaetes/chlamydia, are more distantly related and share sequence identities in the range of only 20% (*e.g.* from *Amycolatopsis methanolica* (16) or *Streptomyces coelicolor* (25) as well as from the Archaeon *T. tenax* (15, 26). Accordingly, they form additional main clusters within the PFK-A family. Within the 40 kDa “short” PFK-As, ADP-PFKs form a distinct cluster divided into ADP-PFK-A1s and ADP-PFK-A2s, including Sll1196 (UniProt P72830) and Sll0745 (UniProt Q55988), sharing 43% sequence identity. The ADP-PFK-As appear more closely related to the short PP_i_-PFK-As than to the classical, short ATP-PFK-As indicating a common origin of both subgroups. This overall tree topology has also been observed in previous studies (14, 26, 27, 28, 29) but the clustering of cyanobacterial PFK-As were poorly resolved and the phosphate donor specificity of the homologs (herein shown to use ADP as phosphate donor) has never been reported before.

**Figure 6:**
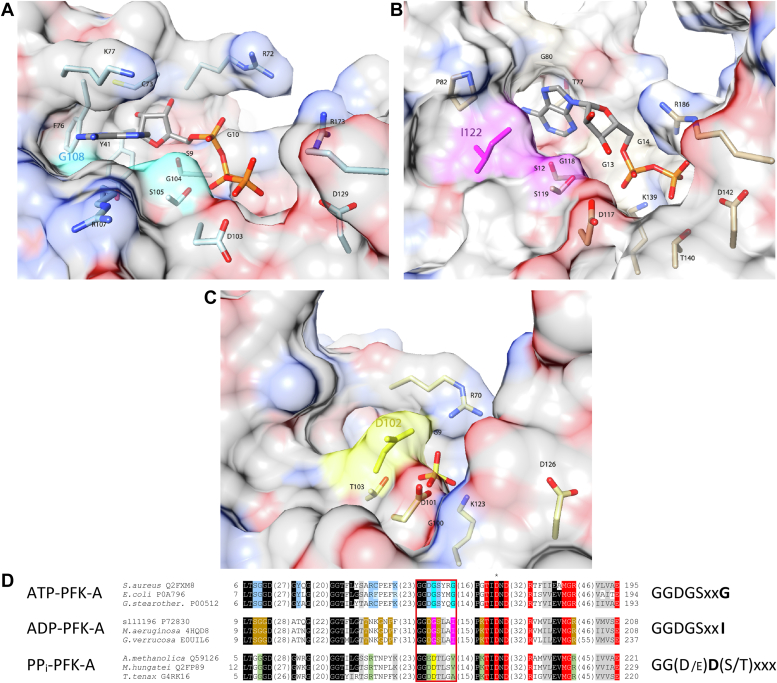
**Elucidation of specific phosphate donor binding motifs in the PFK-A family.** Comparison of the ATP binding site in the ATP-PFK-A from *S. aureus* (*A*) with the proposed ADP-binding site in the ADP-PFK-A1 (Sll1196) from *Synechocystis* (*B*) and the phosphate binding in the PP_i_-PFK-A from *T. tenax* (*C*). The sulfate ion shown in the latter structure was indicated to adopt the same position as the non-transferred phosphate of pyrophosphate in the *Borreliella burgdorferi* PP_i_-PFK (33, 34) and superimposes well with the *β* phosphate of ATP in *e.g.* the *S. aureus* ATP-PFK-A. Proteins are depicted as transparent surface representations (*A*–*C*, colored according to element). ATP, ADP, and sulfate as well as the residues involved in phosphate donor binding are labeled and shown as stick models. Together with the condensed sequence alignment shown in (*D*) these structures illustrate the similarities as well as the differences between ATP-, ADP-, and PP_i_-PFK-As. Of note, those residues, *i.e.* I122 in Sll1196 and D102 in the PP_i_-PFK-A from *T. tenax*, preventing ATP binding are integral constituents of the (putative) ADP and PP_i_ binding pockets. In the condensed alignment (*D*), the key residues for the different phosphate donor specificities discussed in the text and also shown in the panels A-C are highlighted in *cyan* (ATP-PFK-A), *pink* (ADP-PFK-A), and *yellow* (PP_i_-PFK-A). Additional residues involved in ATP binding in ATP-PFK-As are shown in *blue font*, ADP binding in ADP-PFK-As in *brown*, phosphate binding in PP_i_-PFK-As in *green*, and F6P binding are shown in *red*. To the right of the sequences the signature motifs defining the ATP, ADP, and PP_i_ dependence are indicated (based on the *red* outlined part of the alignment). Furthermore, the catalytically essential aspartate is shown in the structures (*A*–*C*) and indicated by an asterisk in the alignment.

The F6P binding site (as in bacterial ATP-PFK-A structures) and the catalytically essential aspartate (D127 in the *Escherichia coli*) are highly conserved throughout the PFK-A family (30, 31, 32, 33) including the cyanobacterial ADP-PFKs (see alignment Fig. S6). This suggests that the catalytic site and phosphate transfer mechanism are similar for ADP- and ATP-PFK-As. However, this requires that the position of the phosphate moiety to be transferred is similar in all PFK-As but in turn necessitates a different binding mode of the adenine moiety in ADP-PFKs compared to ATP-PFK-As (Fig. 6, Fig. S8). Accordingly, the cleft for adenine binding formed by two glycine residues in ATP-PFKs (30) (G108 and G104, in Figs. 6*A*, S9, *A* and *D*), is partly obstructed in ADP-PFK-As by space-filling, hydrophobic isoleucine replacing the first glycine, while the second glycine important for binding of the α phosphate is conserved in ADP-PFK-As (G118) and opens up an alternative binding pocket for the adenosine moiety (Figs. 6*B*, S9, *B* and *E*). In the PP_i_-PFK-As, the second glycine (G104 *Staphylococcus aureus*; G118 Sll1196, Fig. 6, *A* and *B*) is replaced by a conserved negatively charged aspartate residue (D102 in the *T. tenax* enzyme), acting as an anchor for PP_i_ binding but preventing binding of the adenine moiety (15, 33) (Fig. 6*C*, S8, *C* and *F*). Mutating this aspartate residue to glycine in the PP_i_-PFK-A from *Entamoeba histolytica* altered phosphate donor specificity from PP_i_ to ATP (34). These conserved differences in the phosphate donor binding site allowed for deducing signature motifs to distinguish between the specificities of PFK-As for ATP, ADP, or PP_i_ as indicated in Figs. 6*D* and S8*G*.

Additional structural comparisons of ATP-PFK-As and ADP-PFK-As indicated differences in their regulatory properties. Typically, bacterial ATP-PFK-As are allosterically regulated by ADP and PEP, with the allosteric binding site located at the dimer interface (32, 35). This allosteric binding site appears to be blocked by a conserved aspartate residue in ADP-PFK-A1 (D64, in Sll1196, Fig. S6), and it is unknown whether 3PG, the allosteric regulator (inhibitor) of the ADP-PFK-A1, can bind to this site. In ADP-PFK-A2, which exhibits allosteric inhibition by ATP, the effector binding site appears to be shielded by a loop insertion (L69-D76, Sll0745, Fig. S6), preventing access for small molecules.

To confirm our phylogenetic and structural (alphafold) analyses, we tested the co-substrate specificity in several cyanobacteria and alphaproteobacteria that we predicted to be ADP-dependent. For two cyanobacterial PFK-As, from *Microcystis aeruginosa* (PFK-A2) and *Crocosphera subtropica* (PFK-A1), as well as for PFK-A1 from the alphaproteobacterium *Azospirillum brasilense* soluble protein expression was achieved. The proteins were purified *via* IMAC (Fig. S9*A*) and all three tested enzymes were specific for ADP and showed no activity with ATP (Fig. S9*B*), in agreement with our prediction.

## Discussion

Carbon metabolism in *Synechocystis* is notoriously complicated, involving four glycolytic pathways (EMP, ED, OPP, and PK), and the CBB pathway for inorganic carbon assimilation (see Figs. 1 and S11 for a pathway overview). Under photoautotrophic conditions, the EMP pathway must operate in anabolic direction as part of the CBB cycle (36), and under heterotrophic conditions, the EMP runs in the catabolic direction (37), but during photomixotrophic growth the direction is not pre-defined, and appears to be dependent on CO_2_ and light availability (1, 38), reflecting the relative contributions of carbon assimilation and dissimilation reactions, which are highly intertwined (2). Flux analysis methods have been essential to disentangle the contributions of the different pathways (1). The directionality of the EMP is dependent on the environmental conditions, which can change rapidly, and therefore we studied the role of allosteric regulation of the irreversible PFK reaction in enabling this flexibility. Transcriptional analyses and protein abundance for many metabolic enzymes (including PFK and FBPase) did not show large variations between photoautotrophic, photomixotrophic, and heterotrophic growth (3). With both the PFK and the FBPase simultaneously expressed, strict regulation of their activity is essential to prevent futile cycling and should be in accordance with their functional role in metabolism.

One of the two FBPase isoenzymes has been characterized and the structure has been elucidated (9, 10). The F-I enzyme (*slr2094*) is bifunctional, possessing FBPase (class 2) and sedoheptulose 1,7-bisphosphatase (FBP/SBPase) activities. Mg^2+^ and AMP bind competitively to the allosteric site, which is affected by the reduction state of the ferredoxin/thioredoxin system (10). Analysis of the regulation of the FBP/SBPase is not straightforward as it is hard to estimate the contributions of the reduction state and Mg^2+^ on AMP inhibition *in vivo*, but both ATP and NADPH favor the enzyme activity, and both are likely to be high under photosynthetic conditions.

We characterized the two *Synechocystis* PFK isoenzymes, which both use ADP exclusively as phosphate donor, and exhibit hyperbolic saturation curves for their substrates in the absence of their allosteric regulators 3PG (PFK-A1), or ATP (PFK-A2), but in the presence of their regulator exhibit sigmoidal saturation curves. While inhibition of PFK-As by ATP is frequently observed (*e.g.* in yeast (39)), inhibition by 3PG, is not widespread. It has been reported in a number of photosynthetic organisms, for the ATP-PFK from the green alga *Dunaliella marina* and *Selenastrum minutum* located in the chloroplast (40, 41) as well as the plant plastid and cytosolic ATP-PFK from *Ricinus communis* L (42), but also for rabbit ATP-PFK C (43).

To test whether the allosteric regulation of ADP-PFK-As in *Synechocystis* can account for the observed flux distributions, we calculated the percentage activity of the iso-enzymes at metabolite concentrations obtained in a metabolomic study under photoautotrophic and photomixotrophic conditions (44). These concentrations (calculated essentially as in (48), see SI for details) resulted in an effective inhibition of PFK-A1 and A2 under photoautotrophic conditions, with respectively 0.16% and 16.5% of maximal activity, and high activity under photomixotrophic conditions (92% and 86%). Note that in (49), where the EMP runs in an anabolic direction under photomixotrophic conditions, higher 3PG, and lower F6P concentrations were measured, leading to much less favorable conditions for the PFK.

The allosteric regulation appears suitable to switch off the PFK-A isoenzymes during carbon assimilation (high 3PG concentrations, typically 3–5 mM, inhibiting PFK-A1) in the light period, (high ATP production during photosynthesis, inhibiting PFK-A2), and the inhibition can be overcome by increased concentrations of F6P (likely during heterotrophic metabolism) or ADP (during the dark period). The ADP dependency of the PFK is a double insurance that the enzyme is only active under low-energy state conditions. Switching between the hyperbolic and sigmoidal kinetics as a function of the allosteric regulator allows for strong changes in activity with relatively small changes in substrate concentrations, but more quantitative metabolomics and flux data are necessary to test our hypothesis more strictly, in particular, to simulate the photomixotrophic dependency on available CO_2_ and light.

Knockout studies in *Synechocystis* showed that double *pfk* mutants (Δ*pfk1,2*) had no growth phenotype under photoautotrophic or photomixotrophic growth conditions (1). However, reduced growth of the mutant compared to the wild type was observed when glucose was provided in the dark (2, 5). Moreover, a severe effect of Δ*pfk1,2* on polyhydroxybutyrate (PHB) production was reported (50), with the EMP pathway playing an important role in pyruvate and PHB production, particularly under dark or limited oxygen conditions.

Sequence similarity and structural (alphafold) analyses revealed that the *Synechocystis* ADP-PFKs belong to the PFK-A family and adopt its characteristic fold, in contrast to the ribokinase fold (PFK-B family) observed in ADP-PFKs primarily found in archaeal species (Fig. S7). This differentiation underscores the evolutionary diversity in the PFK-A family and broadens the spectrum of phosphate donors to include ADP alongside ATP and PP_i_. Moreover, our phylogenetic analysis which included sequence sets used in previous studies of the PFK-A superfamily (14, 15, 26, 27, 28, 29) demonstrated that ADP-PFK-As form a monophyletic group of “short” 40 kDa PFK-As, alongside ATP-PFK-As from bacteria and PP_i_-dependent PFK-As from Bacteria and Archaea. This clustering reflects their common ancestry and reveals the existence of distinct phosphate donor specificities among PFK-As. It has been previously noted that the change in phosphate donor specificity likely occurred independently several times in different subgroups of PFK-A enzymes, which also applies to the allosteric effector specificity ((14, 51, 52), and literature therein). In accordance with that, the “short” PP_i_-PFK-A cluster also contains the ATP-PFK from *S. coelicolor* (53), which might indicate a later diversification of the substrate specificity within Streptomycetes. Additionally, ADP-PFK-A homologs were found almost exclusively in cyanobacteria and a few alphaproteobacteria, emphasizing the specific distribution of ADP-PFK-As in certain bacterial lineages. Furthermore, the ADP-PFK from cyanobacteria and alphaproteobacteria appeared to be closer related to the “40 kDa” PP_i_-PFK-As than to the ATP-PFKs. Conversely, all plant PFK enzymes are found in a separate PFK-A clade of large enzymes making it very unlikely that the cyanobacterial PFKs are the direct ancestors of plant PFKs. This tree topology was also observed before (28) suggesting that the 40 kDa ADP-PFK-As might have originated from PP_i_-PFK-As.

The clustering of PFK enzymes on the basis of phosphate donor dependency was validated by confirming the co-factor dependency of PFK isolated from *C. subtropica, M. aeruginosa*, and *A. brasilense*, for which ADP dependency was predicted on basis of the clustering. Herewith we could correct the exclusion of the EMP pathway in *A. brasilense*, which was based on the absence of ATP-PFK activity in crude extracts (54). In addition, our findings suggest that *Rhodospirillum rubrum*, a facultative photosynthetic purple non-sulfur bacterium with biotechnological relevance, utilizes ADP-PFK-A rather than ATP-PFK-A for fructose fermentation (55).

The F6P binding site in PFK-As is highly conserved, indicating a shared catalytic mechanism. However, the binding mode for the adenine moiety in ADP-PFKs differs from that in ATP-PFK-As and that of PP_i_ in PP_i_-PFK-As, involving a unique cleft and specific residues, allowing for the respective phosphate donor specificity. The derived consensus motifs for ATP-PFK-As is GGDGSxxG, for ADP-PFK-As GGDGSxxI, and for PP_i_-PFK-As GG(D/E)D(S/T)xxx (with the residues that interfere with other phosphate donor binding shown in bold).

## Conclusions

We have resolved the unclarity on the classification of PFK in *Synechocystis* and have conclusively shown that the two iso-enzymes belong to the PFK-A superfamily, thereby defining a new class of “small” ADP-dependent PFK-As. The grouping includes several other cyanobacteria and also some alphaproteobacteria for which we could predict and confirm ADP dependency. Conserved differences in the phosphate donor binding site allowed for deducing signature motifs to distinguish between the specificities of PFK-As for ATP, ADP or PP_i_. The allosteric inhibition of catabolic PFK-A1 and PFK-A2 by 3PG and ATP, respectively, in conjunction with the inhibition of anabolic F/SPBase by AMP and activation by reducing conditions allow for precise control over glycolysis and gluconeogenesis (see Fig. S11 for a metabolic pathway overview), preventing futile cycling and optimizing carbon utilization in photosynthetic cyanobacteria.

## Experimental procedures

### Gene cloning and protein overexpression

The PFK-A1 and PFK-A2 encoding genes *sll1196* and *sll0745* were amplified from *Synechocystis* sp. PCC 6803 genomic DNA using the primer sets:

*Sll1196*_forward_NdeI_5′- ACTCAGCATATGGGGGAAATTAAACGC,

*Sll1196*_reverse_BamHI_5′- CACTTCGGATCCTTAATCGTTACCAAGGC,

*Sll0745*_forward_NdeI_5′-CCATGTCATATGGGCACAAAACGTATTG,

*Sll1196*_reverse_BamHI_5′-CTTGCTGGATCCTTAGTCTTCTCCTAG.

(restriction sites underlined).

The PCR products were cloned into the expression vector pET15b (Novagene), respectively. Successful cloning was confirmed by DNA sequencing (LGC genomics, Berlin). For expression, the respective plasmids were transformed into the *E. coli* strain Rosetta (DE3, Stratagene) and overexpression was performed in 2 L LB medium containing 100 μg/ml ampicillin and 30 μg/ml chloramphenicol. Cells were incubated in a shaker at 37 °C and 180 rpm to an OD_600_ of 0.6 to 0.8 and protein expression was induced with 0.5 mM isopropyl-β-D-thiogalactopyranoside (IPTG). After induction, the cultures were further incubated at 18 °C and 180 rpm for 18 to 22 h. Cells were harvested by centrifugation (15 min, 8630*g*, 4 °C) and the pellets were stored at −70 °C until use.

For the other PFK-A genes, *i.e.* PFK-A2 from *M. aeruginosa* PCC 9808 (WP_002792444.1), PFK-A1 from *Crocosphaera subtropica* (WP_009545916.1) and PFK-A1 from *A. brasilense* (WP_145679549.1), the expression vectors (pET15b) were purchased codon optimized for expression in *E. coli* from BioCat GmbH (Heidelberg, Germany). The plasmids were transformed into *E. coli* Rosetta (DE3, Stratagene) and heterologous expression were conducted in 0.5 L LB medium as described above (induction at OD_600_ 0.5–0.6 with 0.5 mM IPTG, 18–22 h overnight, 180 rpm).

### Protein purification

Due to the cloning strategy, both the recombinant PFK-A1 and PFK-A2 contain an N-terminal 6x histidine-tag, allowing protein purification by immobilized metal ion affinity chromatography (IMAC) using Ni-TED (nickel (tris(carboxymethyl)ethylene diamine) columns (Macherey-Nagel).

For PFK-A1 frozen cells (3.2 g wet weight) were resuspended in 9.6 ml 1x LEW buffer (Macherery-Nagel, 50 mM NaH_2_PO_4_, 300 mM NaCl, pH 8.0) including additional 3 mM dithiothreitol and 10% (v/v) glycerol. The cells were disrupted by passing four times through a French pressure cell (Thermo Scientific, French Pressure Cell Press) at 150 MPa. Cell debris was removed by centrifugation (45 min, 21130*g*, 4 °C) and the histidine-tagged proteins were purified from the supernatant using Ni-TED gravity flow columns according to the manufacturer's instructions, except for the 1x LEW buffer and one x elution buffer was supplemented with 3 mM dithiothreitol and 10% (v/v) glycerol. The elution buffer also contained 700 mM arginine which was necessary to prevent enzyme precipitation. After elution, the enzyme was stored at −70 °C until further use for enzyme assays. The stored enzyme was stable for at least 6 months.

For PFK-A2 the frozen cells (4.8 g wet weight) were first resuspended in 14.4 ml 1x LEW buffer (Macherey-Nagel) containing 3 mM dithiothreitol and disrupted by sonication on ice for 3 × 5 min (1 min interval in between). Cell debris was removed by centrifugation (45 min, 21130*g*, 4 °C) and the histidine-tagged proteins were purified from the supernatant using Ni-TED gravity flow columns according to the manufacturer's instructions, except for that the involved 1x LEW buffer and 1x elution buffer were supplemented with 3 mM dithiothreitol. Elution fractions containing the recombinant proteins were collected and concentrated using centrifugal concentrators (Vivaspin20, Satorius Stedium Biotech, cut-off size 10 kDa). Afterward, the concentrated protein sample was applied onto a size exclusion chromatography column (HiLoad 16/600 Superdex 200 prep grade, Cytivia) pre-equilibrated with 50 mM HEPES/NaOH (pH 7.5, RT) containing 300 mM NaCl and 3 mM dithiothreitol. Proteins were eluted with the same buffer at a flow rate of 1 ml/min (Äkta purifier FPLC system, GE Healthcare). Protein fractions containing PFK-A2 were confirmed by activity measurements and SDS-PAGE. Pure fractions were combined and stored at −70 °C in the presence of 25% (v/v) glycerol.

For the purification of PFK-As from other selected cyanobacteria and alphaproteobacteria (*e.g. M. aeruginosa* PFK-A2, *C. subtropica* PFK-A1, and *A. brasilense* PFK-A1) frozen cells were resuspended in 1xLEW buffer (1 g cells (wet weight)/3 ml buffer) and disrupted by using the French press (see above). Cell debris was removed by centrifugation (45 min, 21130*g*, 4 °C) and the histidine-tagged proteins were purified from the supernatant using IMAC (Ni-TED gravity flow columns) according to the manufacturer’s instructions. Elution fractions were collected and tested for enzyme activity with ATP and ADP. For further use, the enzymes were stored with 10% (v/v) glycerol at −70 °C.

The protein concentration was determined using a modified Bradford assay according to (56) with BSA (Merck) as standard.

### Determination of the native molecular mass

To determine the native molecular mass of the recombinant enzymes, a calibration curve was generated with five proteins (carbonic anhydrase (29 kDa), ovalbumin (43 kDa), conalbumin (75 kDa), aldolase (158 kDa) and ferritin (440 kDa)) from the LMW and HMW gel filtration calibration kits (GE Healthcare). The size exclusion chromatography was performed using the same settings as for the purification of PFK-A2. For PFK-A1 5 mg/ml (62.5 μM) and for PFK-A2 7 mg/ml (87.5 μM) were applied. The native molecular mass of PFK-As was then calculated using the generated calibration curve.

### Native MS analysis

#### Sample preparation

For native MS analysis, purified PFK-A1 and PFK-A2 had to be rebuffered in advance to 200 mM or 1 M ammonium acetate buffer (NH_4_OAc; pH 6.8; Sigma-Aldrich, A2706; diluted in MS water, Honeywell, 14,263). The corresponding rebuffering procedure involved concentrating the protein solution and then diluting it with the corresponding MS-compatible buffer using 10 kDa molecular mass cut-off spin-filter columns (UFC501096, Millipore) (57). For PFK-A1, three rebuffering cycles were while for PFK-A2, six rebuffering cycles were applied. The resulting protein concentration was subsequently quantified *via* microvolume spectroscopy (DeNovix, DS-11+) and adjusted to a 5 μM stock concentration in the 200 mM or 1 M NH_4_OAc buffer.

#### Native mass spectrometry

The samples were ionized using a TriVersa NanoMate nanoESI system (Advion) equipped with 5 μm diameter nozzle spray chips (Advion, HD_A_384). For each measurement, a 5 μl sample volume was picked out from a well containing the corresponding protein solutions that were previously aliquoted into 96-well plates. The ESI spray was generated using 0.8 psi nitrogen backpressure combined with a positive nozzle chip voltage of 1.7 kV with spray sensing turned on (15 s threshold). MS spectra were recorded for 2 minutes in positive EMR mode on an Exactive Plus EMR Orbitrap mass spectrometer (ThermoFisher) previously calibrated with CsI (2 mg/ml, Thermo Scientific, 192,820,010). The corresponding MS parameters are summarized in Table S1.

#### Data analysis

UniDec software (version 6.0.4) was used for mass deconvolution using standard settings except those listed in table Y. GraphPad (version 8.0.1) was used for data visualization (Table S2).

### Enzyme assays

PFK activity was determined in a coupled assay by following the formation of fructose 1,6-bisphosphate (FBP) using FBP aldolase (FBPA), triosephosphate isomerase (TPI) and α-glycerolphosphate dehydrogenase (GPDH) as auxiliary enzymes. The produced FBP is first cleaved by FBPA to glyceraldehyde-3-phosphate (GAP) and dihydroxyacetone phosphate (DHAP), GAP is then converted to another molecule of DHAP by TPI and finally, DHAP is reduced to glycerol-3-phosphate by GPDH accompanied by the oxidation of NADH to NAD^+^. Thus, two molecules of NADH are oxidized per molecule of FBP produced. The oxidation of NADH was followed as a decrease of absorption at 340 nm and 30 °C (extinction coefficient of NADH = 6.22 mM^-1^ cm^-1^) using a spectrophotometer (SPECORD 210, Analytik Jena GmbH, PFK-A1) or a microplate reader (Infinite M200, TECAN, PFK-A2; calibration curve 0-0.7 mM NADH for quantification). One unit (1 U) of enzyme activity is defined as 1 μmol of product (FBP) formed per minute.

The standard assay mixture (final volume of 500 μl for PFK-A1 and 200 μl for PFKA-2) contained 0.1 M HEPES/NaOH (pH 7.7, 30 °C), 1 U/ml FBPA from rabbit muscle (Merck), 10 U/ml TPI from rabbit muscle (Merck), 1 U/ml GPDH from rabbit muscle (Merck), 0.2 mM NADH and 10 mM MgCl_2_ for PFK-A1 (2.0–2.5 μg enzyme used), and 0.7 mM NADH, 5 mM MgCl_2_ for PFK-A2 (0.74 μg enzyme used). To address phosphate donor specificity, assays were performed with 1 mM F6P in the presence of 1 mM ATP, ADP or pyrophosphate (PP_i_). For the saturation curves with F6P and ADP for PFK-A1 3 mM ADP and 0 to 1 mM F6P as well as 1 mM F6P and 0 to 3 mM ADP and for PFK-A2 3 mM ADP and 0 to 1 mM F6P as well as 0.8 mM F6P and 0 to 6 mM ADP were used. For effector studies non-saturating conditions (*i.e.* 0.08 mM F6P and 0.4 mM ADP for PFK-A1, 0.07 mM F6P and 0.2 mM ADP for PFK-A2) were used. Initial effector studies were performed in the presence of 1 mM phosphoenolpyruvate (PEP), citrate, 3-phosphoglycerate (3PG), 2-phosphoglycerate (2-PG), DL-malate, AMP, ATP or NaP_i_ (pH 7.7, made by NaH_2_PO_4_ and Na_2_HPO_4_). Negative controls were performed omitting either enzyme or one of the substrates.

To study the inhibition of 3PG on PFK-A1, different combinations of F6P and ADP (0.1 mM F6P and 0.15 mM ADP; 0.08 mM F6P and 0.4 mM ADP; 0.4 mM F6P and 0.4 mM ADP; 3 mM F6P and 0.15 mM ADP; 3 mM F6P and 0.5 mM ADP; 3 mM F6P and 1 mM ADP) in presence of 0 to 2 mM 3PG were tested and the saturation curves were repeated in presence of 1 and 2 mM 3PG. To study the inhibition of ATP on PFK-A2, different combinations of F6P and ADP (0.07 mM F6P and 0.2 mM ADP; 0.05 mM F6P and 0.5 ADP; 0.25 m F6P and 0.5 mM ADP; 0.8 mM F6P and 1 mM ADP; 0.8 mM F6P and 3 mM ADP; 0.8 mM F6P and 5 mM ADP) in presence of 0 to 2 mM ATP were tested and the saturation curves were repeated in presence of 1 and 2 mM ATP.

To address phosphate donor specificity for the other selected ADP-dependent PFK-As, assays were performed under the same conditions as for PFK-A1 (500 μl Volume, 0.1 M HEPES/NaOH (pH 7.7, 30 °C), 0.2 mM NADH, 10 mM MgCl_2_, 1U/ml FBPA, 10 U/ml TPI, 1 U/ml GPDH) with 1 mM F6P and 2 mM ADP or ATP.

### Bioinformatic analysis and computational analysis

Structural models were retrieved from the AlphaFold Protein Structure database (45, 46) or predicted using the ColabFold software (58). SwissDock was used to infere the putative ADP binding sites in *Synechocystis* ADP-PFK-A1 (Sll1196) and ADP-PFK-A2 (Sll0745) (59). Structural analyses, comparisons, and visualizations were done using UCSF Chimera package from the Resource for Biocomputing, Visualization, and Informatics at the University of California, San Francisco (supported by NIH P41 RR-01081) (60). For clustalW alignments and phylogenetic tree constructions the MEGA11 software package was used (61) (for details see legend to Fig. 5).

To estimate the kinetic parameters for the PFK-A iso-enzymes we fitted a MWC model (Equation 1) to the experimental data, using the NonlinearModelFit function in Wolfram Mathematica. The MWC model assumes that the enzyme can exist in two states, a relaxed (R) and tense (T) state, and that the distribution between the two states (L0 = T/R) can be described as a function of the allosteric effector α and preference of binding of the substrates to R or T state, (L = L_0_(1 + α) (1 + c σ)). Initially, we fitted the full MWC equation to the data set, but it became apparent that ADP has a very low affinity for the T form, rendering this enzyme form inactive, and reduces the full MWC equation to Equation 1, which was used for the fits shown in Fig. 3, with the parameter values shown in Table 1. The parameters were well identifiable in the data set, except for L0 which was poorly identifiable.

### Calculation of intracellular metabolite concentrations for PFK inhibition during auxotrophic or mixotrophic growth

In the manuscript: Jablonsky *et al.*, 2013 (48), the authors calculate an internal 3PG concentration of 5.39 mM referring to Takahashi *et al.*, 2008 (44). The 3PG concentration listed in Takahashi *et al.* 2008 is 1346 nmol/g FW under photoautotrophic growth, and this gives a conversion of 0.249 ml/g FW. Using the same conversion factor, we calculated concentrations for all metabolites that affect PFK activity under photoautotrophic and photomixotrophic growth, from the Takahashi *et al.* 2008 manuscript (see Table S3).

## Data availability

All study data are included in the article and SI Appendix. In addition, the kinetic data and model simulations shown in Fig. 3 are available as Excel files and Mathematica notebooks on the FAIRDOMHub (https://fairdomhub.org/investigations/660) (doi:10.15490/fairdomhub.1.investigation.660.1).

## Supporting information

This article contains supporting information (30, 31, 32, 33, 34, 35, 44, 45, 46, 47).

## Conflict of interest

The authors declare that they have no conflicts of interest with the contents of this article.
