# Supplementary material for: Structure function analysis of ADP-dependent cyanobacterial phosphofructokinase reveals new phylogenetic grouping in the PFK-A family
Source: J Biol Chem. 2024 Oct 10;300(11):107868. doi: 10.1016/j.jbc.2024.107868 (PMC11609450; doi:10.1016/j.jbc.2024.107868)
Supplement: Supplemental information [file mmc1.pdf]

Supporting Information for

Structure function analysis of ADP-dependent cyanobacterial phosphofructokinase reveals new phylogenetic grouping in the PFK-A family

Lu Shen[1‡], Carmen Peraglie[1‡], David Podlesainski[2], Christina Stracke[1], Ravi Shankar Ojha[1], Frauke Caliebe[3], Markus Kaiser[2], Karl Forchhammer[4], Martin Hagemann[5], Kirstin Gutekunst[3], Jacky L. Snoep[6,7#], Christopher Bräsen[1*], Bettina Siebers[1*]

[1]Molecular Enzyme Technology and Biochemistry (MEB), Environmental Microbiology and Biotechnology (EMB), Centre for Water and Environmental Research (CWE), Faculty of Chemistry, University of Duisburg-Essen, Essen, Germany.

[2]Chemical Biology, Centre of Medical Biotechnology (ZMB), Faculty of Biology, University of Duisburg-Essen, Essen, Germany.

[3]Molekulare Pflanzenphysiologie, University of Kassel, Kassel, Germany.

[4]Microbiology, University of Tübingen, Tübingen, Germany.

[5]Plant Physiology, University of Rostock, Rostock, Germany.

[6]Biochemistry, University of Stellenbosch, Stellenbosch, South Africa.

[7]Molecular Cell Biology, Vrije Universiteit Amsterdam, Amsterdam, The Netherlands.

[#] Contributing authors: jls@sun.ac.za

[‡] These authors contributed equally to this work.

* For correspondence: Christopher Bräsen, christopher.braesen@uni-due.de; Bettina Siebers, bettina.siebers@uni-due.de.

This PDF file includes:

Supporting text
Figures S1 to S11
Tables S1 to S3

Supporting text

Sequence and structural comparison of the ADP-PFK-As with ATP- and PPi-PFK-As

From the sequence alignment shown in Fig. S6 as well as from structural comparisons it becomes evident that the fructose-6-phosphate (F6P) binding site as well as the catalytically essential aspartate (D127 in *E. coli* and D129 in *S. aureus* ATP-PFK-A) acting as a general base in catalysis (31-33) are well conserved in nearly all PFK-As. This indicates that the basic reaction mechanism, the position of the sugar phosphate, and the spatial orientation of the phosphate moiety to be transferred from the phosphate donor to F6P remains the same in all PFK-As including the ADP-PFK-As from cyanobacteria. In the crystal structure of the PP_i-PFK-A from *Borreliella burgdorferi* a sulfate ion has been observed which accommodates the same position as the β phosphate of ADP in the *E. coli* structure (31-33). From this, it has been concluded that the pyrophosphate in PP_i-PFK-As binds in a similar manner as the β and γ phosphates of ATP in ATP-PFK-As. Hence, it appears tempting to speculate that in the ADP-PFK-As, the α and β phosphates occupy the same position as the β and γ phosphates of ATP and the two phosphates of PP_i in ATP-PFK-As and PP_i-PFK-As, respectively. This would however mean that the adenosine moiety of ADP in ADP-PFK-As cannot bind in the same way as observed for the adenosine of ATP/ADP in ATP-PFK-As. As exemplarily shown for the *S. aureus* ATP-PFK-A (pdb 5xz9 (30)) in Figs. 6, S8, the binding of the adenine ring in ATP-PFK-As takes place in a cleft made up by an α helix (D103-E115 in the *S. aureus* enzyme) and a loop (R72-K77). As revealed by structural comparison of the available ATP-PFK-A crystal structures with the Alphafold models of the ADP-PFK-As from *Synechocystis* (55, 56), this cleft is blocked in ADP-PFK-As by a space filling, hydrophobic isoleucine residue (I122 and I126 in ADP-PFK-A1 (Sll1196) and ADP-PFK-A2 (Sll0745), respectively) in the middle of the corresponding α helix (D117-G130 in Sll1196) which is invariant in all ADP-PFK-As (Fig. 6, S6, S8). This would make a similar binding of the adenine ring as observed in ATP-PFK-As impossible. In ATP-PFK-As this position is accommodated by a glycine residue (G108 in the *S. aureus* ATP-PFK-A) or sometimes by alanine keeping the cleft open for adenine binding.

In ATP-PFK-As a second position in the α helix D103-E114 is invariantly occupied by a glycine residue (G104) which also keeps the ATP binding cleft open mainly for the α phosphate of the nucleotide (for explanation see (33)). The glycine in this position is also conserved in ADP-PFK-As (G118 in ADP-PFK-A1 (Sll1196) and G122 in ADP-PFK-A2 (Sll0745)), which presumably leaves the space in the active site open to allow for binding of the adenosine moiety of the ADP phosphate donor (32). Conversely, this position is adopted by a conserved negatively charged aspartate residue in all PP_i-PFK-As (D177 in the *B. burgdorferi* enzyme) totally preventing ADP/ATP binding, both sterically and electrostatically, but providing a kind of anchor to direct and hold the pyrophosphate (lacking the adenosine moiety) in the correct position (33). Notably, mutation of the aspartate residue in the PP_i-PFK-A from *Entamoeba histolytica* to glycine resulted in a change of the phosphate donor specificity from PP_i to ATP (34).

The potential alternative adenosine binding pocket in ADP-PFK-As is shown in Figs. 6, S8 and might involve interactions of I126, P82, G80, T77, S12, and S119 with the adenine ring, as well as of G13 with the ribose moiety. S12, S119, G13, and R186 are also conserved in ATP-PFK-As. R186 appears essential in coordinating both ribose and the phosphate residues. However, S12, S119, G13 fulfil slightly different functions in interacting with the ribose moiety and the α phosphate (30-32) instead of the adenine in ADP-PFK-As. The contacts with the α and β phosphates in the ADP-PFK-As, might presumably be provided by G13, G14, G116, as well as K139, T140, and R186. Nearly all of the latter phosphate interacting residues are also functionally conserved in ATP and PP_i-PFK-As although with slight changes in the interactions

with α-β and β-γ phosphates, respectively. Only, K139 is invariantly substituted in "short" ATP-PFK-As by a glycine residue (G124 in the *S. aureus* and *E. coli* ATP-PFK-A) which might indicate a function also of this position in discriminating between ATP and ADP/PP_i, respectively. However, in "long" ATP-PFK-As from plants, protists and spirochaetes/chlamydia this lysine is also present (instead of a glycine) suggesting some more subtle differences in the coordination of the phosphates. Another residue, i.e. R72 (e.g. in the *S. aureus* and *E. coli* ATP-PFK-A) making contacts to the α phosphate of ATP/ADP (32) is conserved in ATP and PP_i-PFK-As. In ADP-PFK-As it is also substituted (mostly by serine or asparagine) further indicating a different binding mode of the α phosphate as well as the adenosine moiety in ADP-PFK-As. In addition, the two aspartate residues 103 and 131 in the *S. aureus* (D103/D129 in *E. coli*) ATP-PFK-A coordinating especially the β and γ phosphates of ATP via a water/Mg^{2+} network are highly conserved in the whole PFK-A superfamily including ADP-PFK-A1 (D117/D144) and ADP-PFK-A2 (D121/D148) of *Synechocystis* where they likely fulfil a similar function in α-β phosphates coordination of ADP.

In addition, the regulatory properties of the two cyanobacterial ADP-PFK-As may at least partly be explained based on the Alphafold models of the two proteins. In the bacterial ATP-PFK-As allosterically regulated by ADP (activator) and PEP (inhibitor), the effectors bind to the effector binding site located at the dimer interface (32, 35). Although this effector binding site shows some conservation in both ADP-PFK-A1 and A2 (particularly in the residues corresponding to 21/R25/R154 interacting with the (β) phosphate group of ADP and PEP) (Fig. S6), an aspartate residue (D64) conserved in ADP-PFK-A1 (Sll1196) homologues would strongly interfere with PEP and ADP binding in a similar position/orientation as observed in bacterial ATP-PFK-As (32, 35) coinciding with the lack of responsiveness towards these common PFK-A regulators. If 3PG shown to act as allosteric regulator of the ADP-PFK-A1 (Sll1196) nonetheless binds to this site remains to be shown. The conservation of the arginine residues mentioned above might suggest a similar binding mode of the phosphate group of 3PG but the rest of the effector molecule interactions needs then to be different from those observed with PEP. The cyanobacterial ADP-PFK-A2 (Sll0745) did not show allosteric behavior towards the known PFK effectors. In agreement with this finding, the Alphafold model of the *Synechocystis* PFK-A2 dimer suggested that a loop insertion (L69-D76) also clearly visible in the sequence alignment (Fig. S6) likely shields the effector site - which is moreover even less conserved than in *Synechocystis* PFK-A1 – and would completely hamper small molecules from entering this region of the dimer interface.

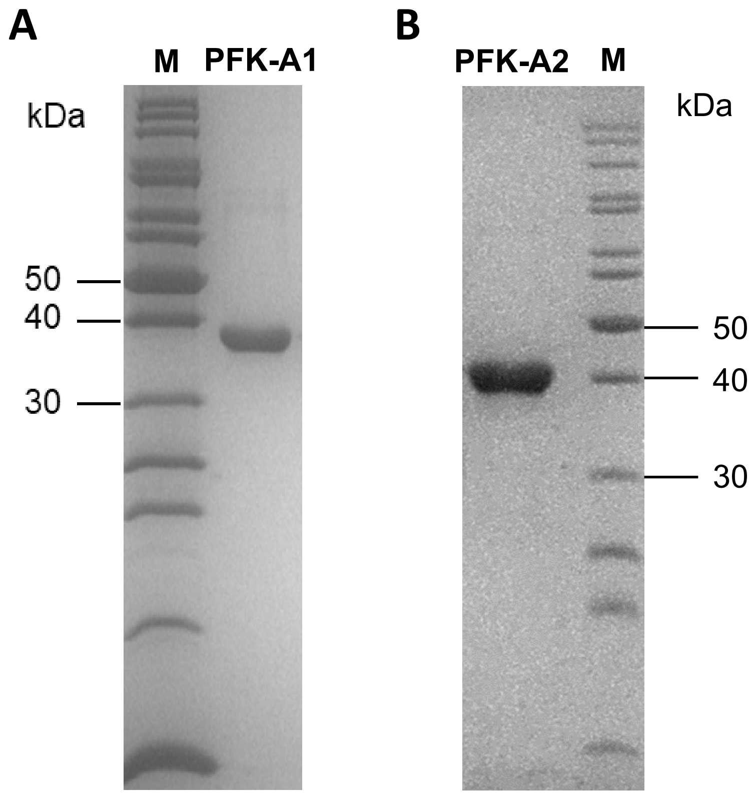

Figure S1. Purification of the recombinant ADP-PFK-A1 (A) and ADP-PFK-A2 (B) from *Synechocystis* sp. PCC 6803. Protein fractions (3 µg) after size exclusion chromatography were separated via SDS-PAGE and gels were stained with Coomassie Brilliant Blue. M, Marker (PageRuler™ Unstained Protein Ladder, Thermo Fischer Scientific).

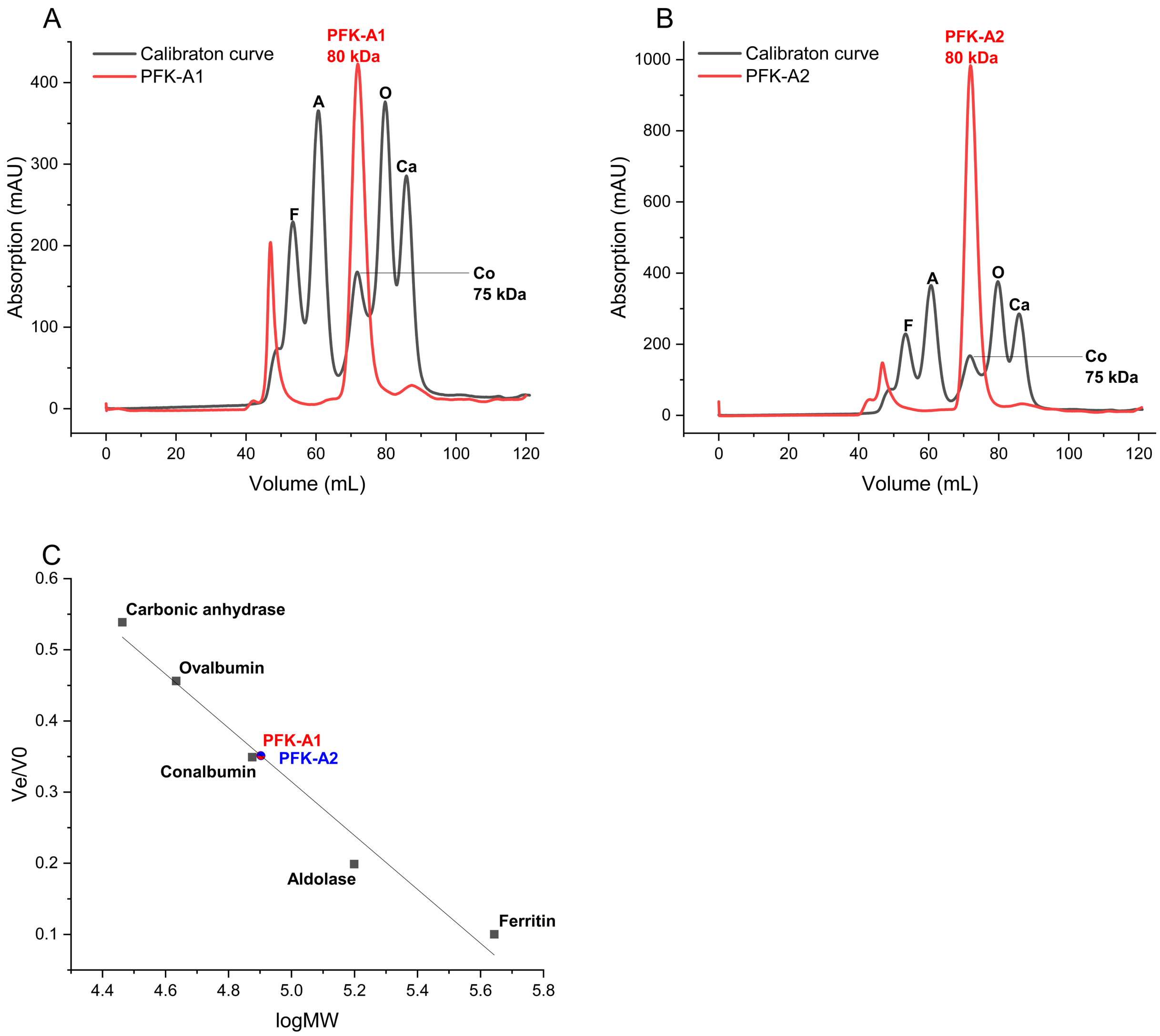

Figure S2. Native molecular mass of ADP-PFK-A1 (A) and ADP-PFK-A2 (B) from *Synechocystis* sp. PCC 6803. The native molecular mass of PFK-A1 and PFK-A2 were determined via size exclusion chromatography (HiLoad 16/600 Superdex 200 prep grade, Cytivia). For PFK-A1 5 mg/ml (62.5 µM) and for PFK-A2 7 mg/ml (87.5 µM) were applied. The calibration curve (grey) was generated with five proteins (carbonic anhydrase (Ca, 29 kDa), ovalbumin (O, 43 kDa), conalbumin (C, 75 kDa), aldolase (A, 158 kDa) and ferritin (F, 440 kDa)) from the LMW and HMW gel filtration calibration kits (GE Healthcare).

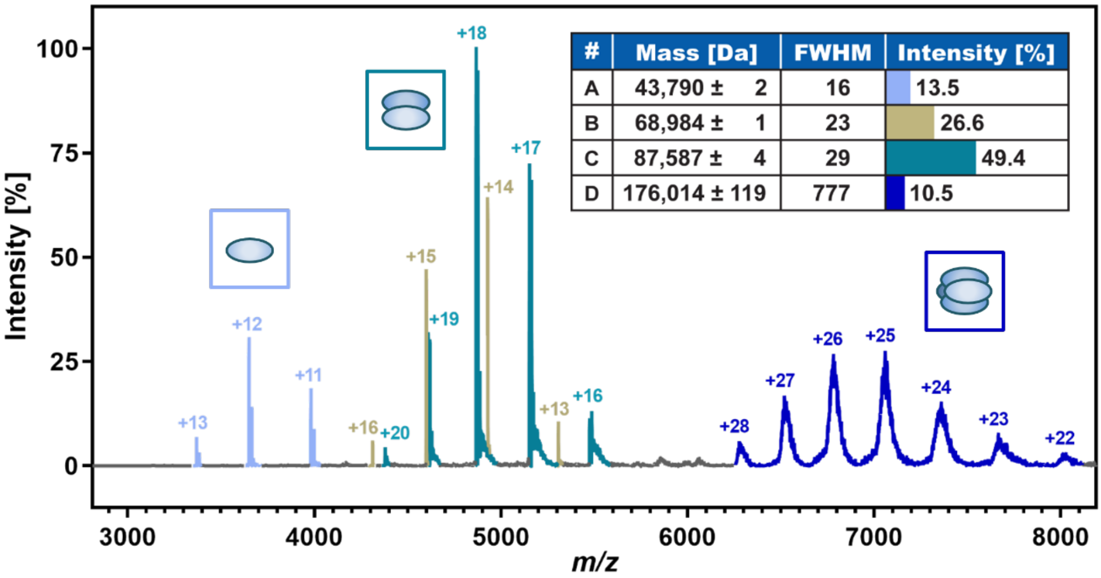

Figure S3. Native MS analysis revealed the presence of different oligomeric forms of PFK-A2 (Sll0745) in the MS buffer. MS spectrum of 5 µM PFK-A2 in 200 mM NH_4OAc is shown as mean of 80 scans with colour coding of signals resulting from the monomeric (light blue), homodimeric (turquoise) and homotetrameric (deep blue) protein form. The deconvoluted protein masses, calculated by UniDec, are provided in the included table along with their standard error, full width at half maximum (FWHM), and relative intensity.

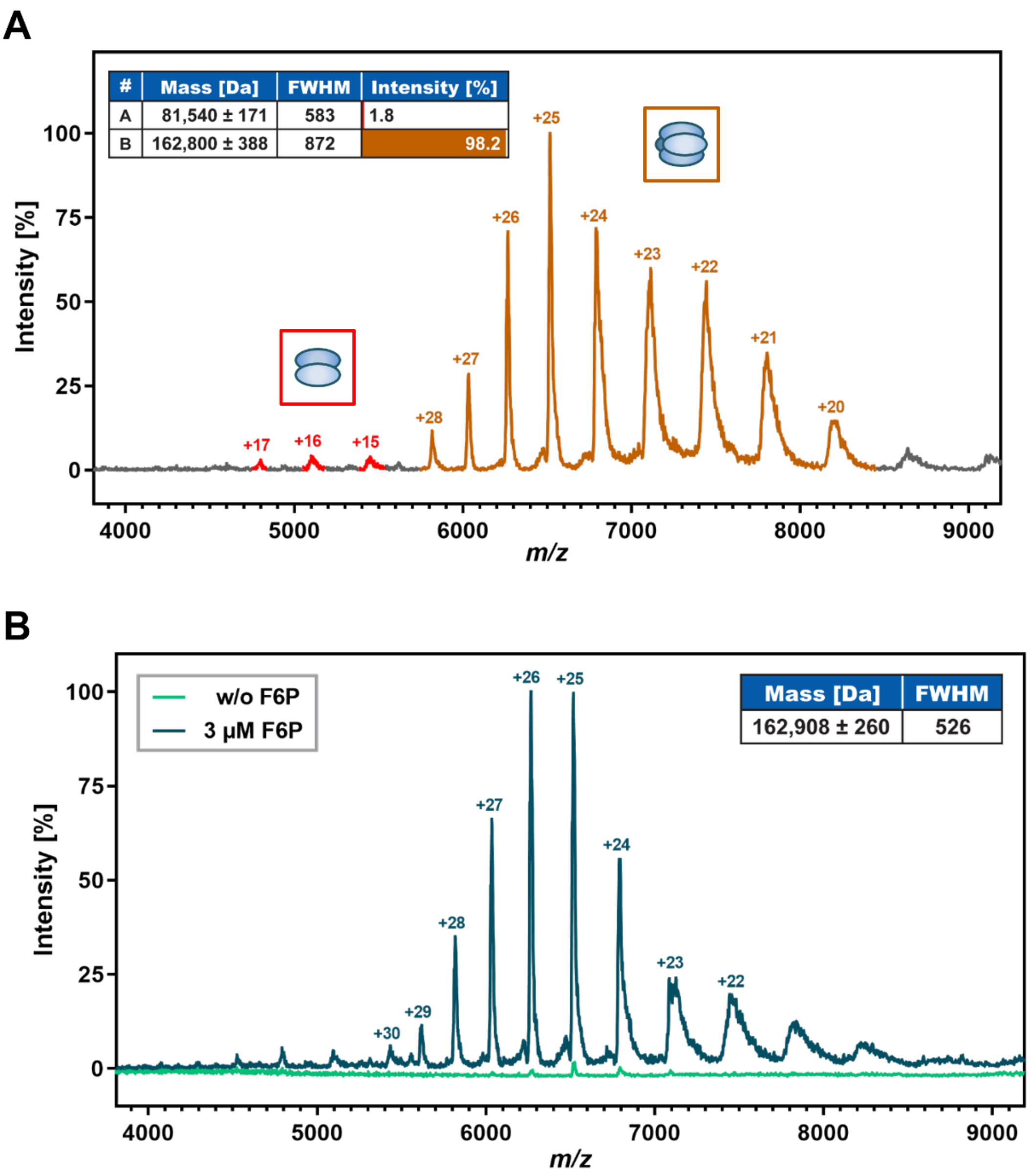

Figure S4. Native MS analysis of PFK-A1 (Sll1196). (A) MS spectrum of 5 µM PFK-A1 in 1 M NH_4OAc is shown as mean of 480 scans with colour coding of signals resulting from the homodimeric (red) and homotetrameric (orange) protein form. The deconvoluted protein masses, calculated by UniDec, are provided in the included table along with their standard error, FWHM, and relative intensity. **(B)** Superposition of MS spectra (mean of 500 scans) of 5 µM PFK-A1 in 200 mM NH_4OAc with (dark green) or without (light green) addition of 3.125 µM F6P.

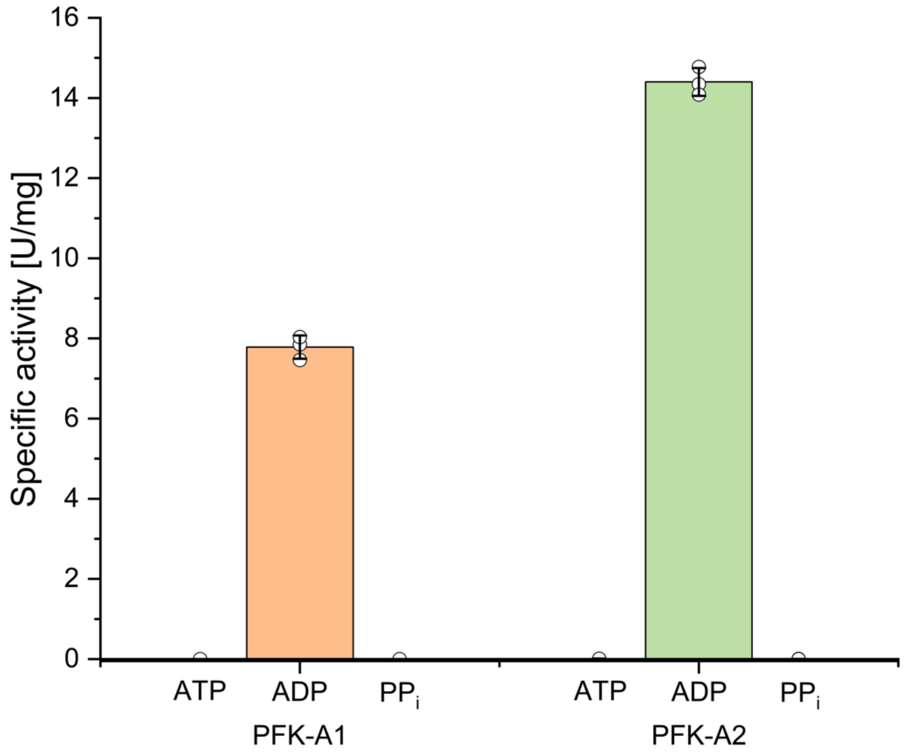

Figure S5. Enzymatic activity of PFK-A1 and PFK-A2 from *Synechocystis* sp. PCC 6803 using different phosphate donors. Assays were performed with 1 mM F6P in present of 1 mM ATP, ADP or pyrophosphate (PP_i). The means and standard deviations for three biological replicates (n = 3) are shown.

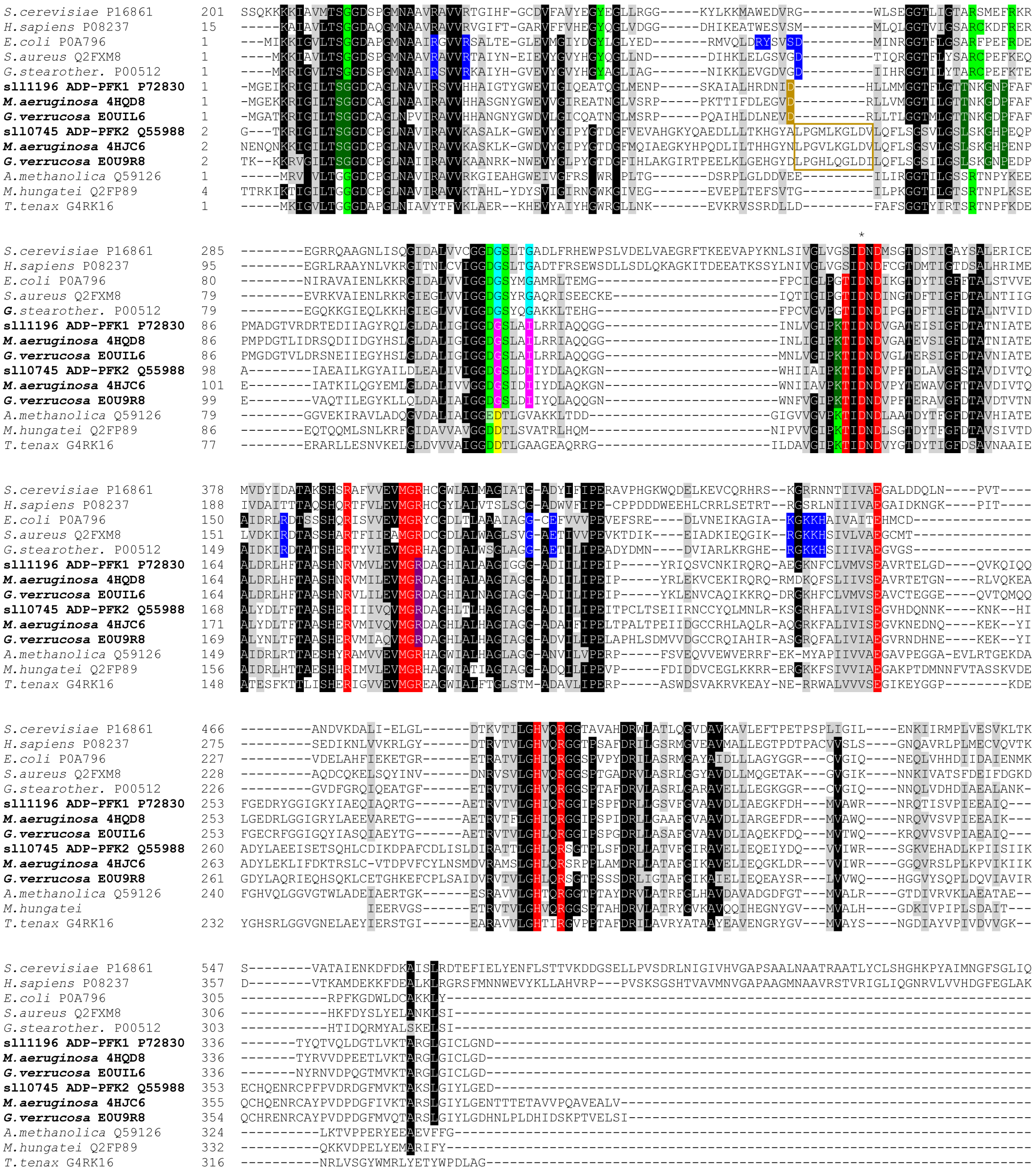

Figure S6. Sequence alignment of *Synechocystis* sp. PCC 6803 ADP-PFK-A1 and ADP-PFK-A2 with other ADP-PFK-A homologues from cyanobacteria and α proteobacteria (highlighted in boldface) as well as with ATP- (upper part) and PP$_i$-dependent PFK-As (lower part) from the "40 kDa subgroup" (see also phylogenetic tree in Fig. 5). The residues predicted to be involved in phosphate donor binding are highlighted in bright green (ATP- and PP$_i$-PFK-As, as shown in the crystal structures from *S. aureus*, *E. coli*, and *G. stearothermophilus* as well as *B. burgdorferi*, respectively) and dark green (ADP-PFK-As) and those involved in F6P binding in red. The residues indicative for the ATP-, PP$_i$-, and ADP-specificity are highlighted in cyan, yellow and magenta, respectively. The arginine residues likely involved in both – (R186) in the ADP-PFK-A1 in *Synechocystis* (Sll1196) – is marked in purple. The aspartate residue acting as general base in catalysis is indicated by an asterisk. Highlighted in blue are those residues forming the effector binding site in ATP-PFK-As for ADP and PEP as shown in the crystal structures from the ATP-PFK-As from *E. coli* of the and *G. stearothermophilus.* The aspartate residue likely interfering with the binding of these effectors to the ADP-PFK-A1 is shown in brown and the loop eventually blocking this part of the dimer interface in ADP-PFK-A2s is boxed in brown.

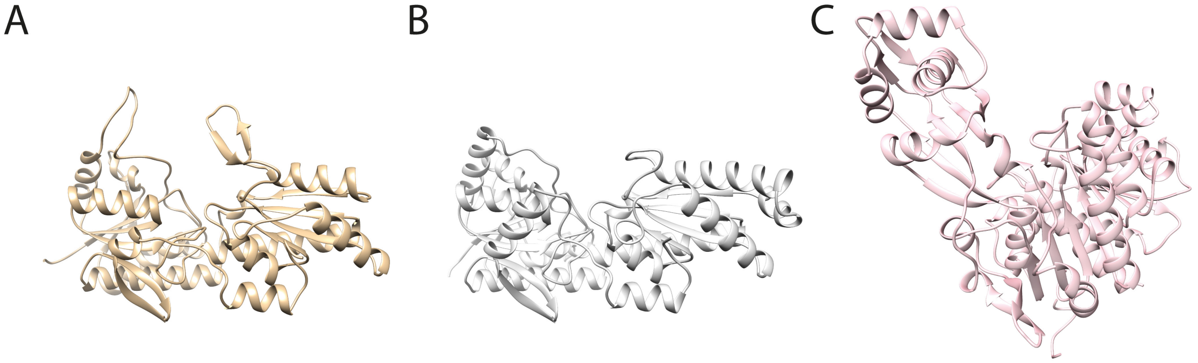

Figure S7. Structural comparison of the *Synechocystis* ADP-dependent PFK-As Sll1196 (ADP-PFK-A1, brown) (A) and Sll0745 (ADP-PFK-A2, grey) (B) with the ADP-dependent PFK from the ribokinase (PFKB) superfamily from *Pyrococcus horikoshii* (3drw, pink) (62) **(C).** The comparison illustrates the different folds of the newly identified cyanobacterial ADP-PFKs and the archaeal ADP-PFK and shows together with the PFK-A superimposition in Fig. 4 that the former not belong to the ribokinase superfamily but rather to the PFK-A superfamily. Monomers of the crystal structure of 3drw as well as the alphafold models of Sll1196 and Sll0745 are shown in ribbon representation.

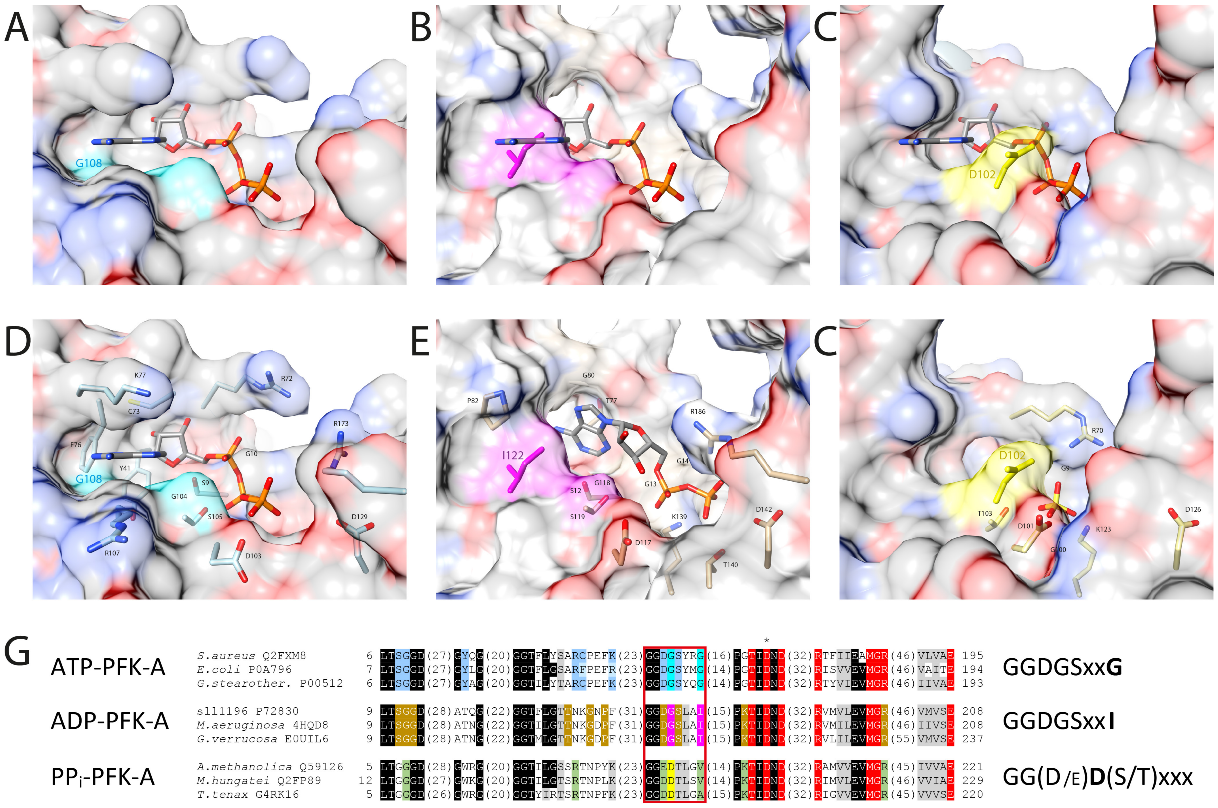

Fig. S8. Structural implications for phosphate donor specificity of ATP-, ADP- and PP$_i$-dependent PFK-As. (A-F) Comparative illustration of the ATP binding site in the *S. aureus* ATP-PFK-A (A, D) with the corresponding regions in the alphafold models of the ADP-PFK-A1 Sll1196 from *Synechocystis* (B, E) and the PP$_i$-PFK from *T. tenax* (TTX_1277) (C, F) demonstrating why ATP cannot bind to the latter two. *S. aureus* ATP-PFK-A, *Synechocystis* ADP-PFK-A1, and *T. tenax* PP$_i$-PFK are shown with corresponding transparent surface (coloured according to element). (A-C) ADP-PFK-A1 and PP$_i$-PFK-A structural models were superimposed on the *S. aureus* ATP-PFK-A (A) and ATP depicted as stick model is shown in the same position in all three structures. In the ATP-PFK-A, ATP is bound in a cleft which is "kept open" by the two glycine residues (G104 and G108 in *S. aureus* ATP-PFK-A) (highlighted in cyan) conserved in ATP-PFK-As (A). This cleft is "blocked" by a conserved isoleucine (I122 in Sll1196, ADP-PFK-A1) invariantly present in all ADP-PFK-As highlighted in magenta which prevents the adenosine moiety from binding in the same manner as in ATP-PFK-As (B). The second glycine (G118 in ADP-PFK-A1 Sll1196, also shown in magenta) is however conserved keeping the space in this position open. Conversely, in PP$_i$-PFK-As this latter position is occupied by a conserved aspartate residue (D102 in the *T. tenax* enzyme, highlighted in yellow) blocking the space for the α phosphate and the ribose moiety, thus preventing ATP and also ADP from binding. To visualize alterations in the specific phosphate donor binding sites for ATP, ADP, and sulfate (D-F) they are presented once more along with the concise sequence alignment (G) (refer to Fig. 6 (main manuscript) for detailed information).

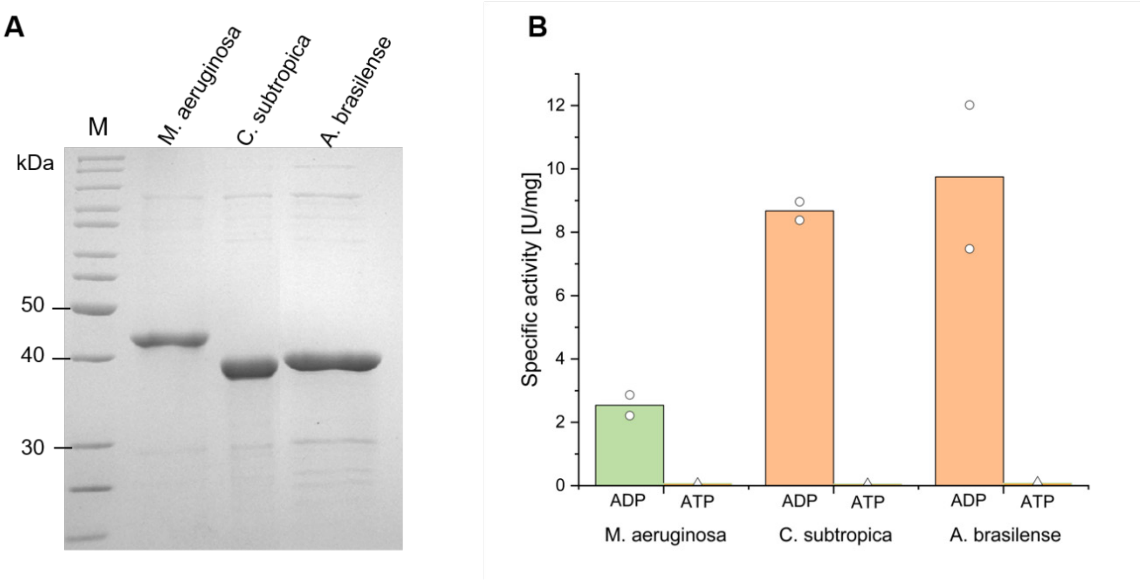

Fig. S9. Phosphate donor specificity of PFK-As from selected cyanobacteria and alphaproteobacteria. The cyanobacterial PFK-As from *Microcystis aeruginosa* (PFK-A2) as well as *Crocosphera subtropica* (PFK-A1) and from the alphaproteobacterium *Azospirillum brasilense* (PFK-A1) were recombinantly expressed in *E. coli*, purified via IMAC and analyzed via SDS-PAGE (A). M, Marker (PageRuler™ Unstained Protein Ladder, Thermo Fischer Scientific). Enzyme activity was determined using 2 mM ADP or ATP as phosphoryl donor (B). The means for two biological replicates (n = 2) are shown.

***Microcystis aeruginosa* PFK-A2 (WP_002792444.1, I4HJC6)**

Codon-optimized DNA sequence

AACGAAAACCAGAATAAGAAGATCGGCATTCTGACCAGTGGTGGCGATTGCCCGGGTCTGAATGCAGTGATTCGC
GCCGTTGTGAAAGCAAGTAAACTGAAAGGTTGGGATGTGTATGGCATTCCGTATGGCACCGATGGCTTCATGCAG
ATTGCAGAAGGCAAATATCATCCGCAGGATCTGATTCTGACCCATCATGGCTATAATCTGCCGGGCGTTCTGAAA
GGCCTGGATGTTCTGCAGTTCCTGAGTGGTAGTGTGCTGGGCAGCCTGAGTAAAGGCCATCCGGAAAATCCGGAA
ATTGCAACCAAAATTCTGCAGGGCTATGAAATGCTGGGCCTGGATGCCCTGATTGTTGTGGGTGGTGATGGTAGT
ATTGATATTATCTATGATCTGGCCCAGAAAGGTAATTGGAATATTATTGCAGTGCCGAAAACCATTGATAATGAT
GTGCCGTATACCGAATGGGCAGTTGGCTTCACCACCGCAGTTGATATTGTTACCCAGGCCCTGTATGATCTGACC
TTCACCGCAGCAAGTCATGAACGCGTTATGATTGTGCAGGTTATGGGCCGTGATGCAGGCCATCTGGCCCTGCAT
GCAGGTATTGCAGGTGGTGCAGATGCAATCTTCATTCCGGAACTGACCCCGGCACTGACCCCGGAAATTATTGAT
GGCTGTTGTCGTCATCTGGCCCAGCTGCGTGCCCAGGGTCGCAAATTCGCACTGATTGTGATTAGTGAAGGCGTG
AAAAATGAAGATAATCAGAAAGAAAAGTACATCGCCGATTATCTGGAAAAACTGATCTTCGATAAAACCAGAAGC
CTGTGCGTGACCGATCCGGTGTTCTGTTATCTGAATAGCATGGATGTTCGCGCAATGAGCCTGGGCCATCTGCAG
CGCAGTCGCCCGCCTCTGGCCATGGATCGTCTGCTGGCAACCGCCTTCGGTATTAAAGCAGTTGAACTGATTGAA
CAGGGTAAACTGGATCGTGTTGTTATCTGGCGCGGTGGTCAGGTTCGCAGCCTGCCGCTGAAACCGGTGATTAAA
ATTATTAAACAGTGCCATCAGGAGAATCGCTGTGCCTATCCGGTGGATCCGGATGGCTTCATTGTTAAAACCGCA
CGCAGTCTGGGTATCTATCTGGGCGAAAATACCACCACCGAAACCGCAGTGGTTCCGCAGGCAGTTGAAGCACTG
GTTTAA

***Crocosphera subtropica* PFK-A1 (WP_009545916.1, B1WQ98)**

Codon-optimized DNA sequence

GGTGAAAAAAAGCGTATTGGCATTCTGACCAGCGGTGGCGATTGCGCCGGTCTGAATGCAGTGATTCGTGCAGTG
GTGCATCATGCAGTTGGCACCTATAATTGGGATGTGATTGGTATTCGCGAAGCAACCCAGGGCCTGATGAGCGAT
CCGCCGCGCTTCATTCAGTTCGAACTGGATAAACTGGATAATCTGCTGCTGATGGGCGGTACCATTCTGGGCACC
ACCAATAAAGGTGATCCGTTCGCATTCCCGATGAATGATGGCACCGTTCGCGATCGCAGCAGCGATATTATTAAT
GGTTATCATAGTCTGGGTCTGGATGCACTGATTGGTATTGGTGGCGATGGCAGCCTGGCAATTCTGCGCCGCCTG
GCCCAGCAGGGCGGTATTAATCTGGTTGGTATTCCGAAAACCATTGATAATGATGTTGGCGCCACCGAAGTTAGT
ATTGGCTTCGATACCGCAACCAATATTGCCACCGAAGCACTGGATCGTCTGCACTTCACCGCCGCAAGCCATAAT
CGTGTGATGATTCTGGAAGTGATGGGTCGCGATGCCGGCCATATTGCACTGGCCGCCGGTATTGCAGGCGGTGCA
GATGTTATTCTGATTCCGGAAATTCCGTATAAAATTGAAAAGGTGTGTCAGAAAATCCGCGCACGCCAGGAAAAA
GGCAAACACTTCTGTCTGGTTATGGTTAGTGAAGCCGTGCGTACCGAAGTTGGTGAACAGGTTAGCAAACTGAAA
CAGTTCGGTGAAGATCGCCTGGGTGGCATTGGTAAATATATTGCAGAACAGATTGGCGCCAAAACCGGTGCCGAA
ACCAGAGTTACCGTGCTGGGCCATATTCAGCGTGGTGGCATTCCGAGTCCGATGGATCGTCTGCTGGGTAGCGCA
TTCGGTGTGGCAGCCGTGGATCTGATTGCCCAGGGTAAATTCGATCAGATGGTTGCCTGGCAGAATCGCCAGATT
CTGAGTGTTCCGATTGCAGAAGCAATTCAGACCTATCGCACCGTTGATCCGGAAGAAACCTTAGTGAAAACCGCA
CGTGGTCTGGGCATCTGTCTGGGCGATTAA

***Azospirillum brasilense* PFK-A1 (WP_145679549.1, A0A560AK87, KAA0679374.1)**

Codon-optimized DNA sequence

ACCGCAGGTAAACGTATTGGTATTCTGACCAGTGGCGGCGATTGTGCAGGTCTGAATGCAGTTATTCGTGCAGTG
GTGCATCGTGCAGTTCTGACCTATGGCTGGCAGGTGCTGGGCATTAAAGAAGGTACCCAGGGTCTGCTGCAGCGT
CCGGTTCAGTATCAGGTTCTGGATCTGCCGCAGGTGGATGGTAATATGATGCGCATGGGTGGTACCATTCTGGGT
ACCACCAATCGTGGTGATCCGTTCGCATATCCGATGGCAGATGGTACCCAGAAAGATCGTAGCGATGAAATTATT
GGCGGTTATCGCGAACTGGGCCTGGATGCCCTGATTGGCATTGGCGGTGATGGTAGCTTCGCCATTCTGAAAAAA
CTGGCCGATAAAGGCGGCTTCCAGATGGTTGGCATTCCGAAAACCATTGATAATGATCTGGGTCTGACCGAAGTG
AGCGTGGGTTATGATACCGCAGTTGGCGTTGCAGTGGAAGCACTGGATCGTCTGCAGCCGACCGCCGCAAGCCAT
GCACGCGTGATGGTTCTGGAAGTTATGGGCCGCGATGCAGGCCATATTGCCCTGGCAGCAGGTATTGCAGGCGGT
GCAGATGTGGTGCTGATTCCGGAAATTGCCTATAGCATTGAAAAAATTGCCCAGAAAATCAAACAGGTGCGCAGT
ACCGGCCGTAACTTCGCACTGGTGGTGGTGAGCGAAGCAGTTAAAACCGTGGATGGTACCGGTGTTCAGAAACTG
TTCCAGGGTGGTCAGAAACGCTATGGCGGCATTGGTGATTATATTGGTGAAAAAATTGCGGATGCAACCGGTGCA
GAAACCAGAGTTACCGTTCTGGGTCATGTGCAGCGTGGCAGCATGCCGAGTCCGCGCGATCGTCTGGTGGCCAGT
GCATTCGGTGTTCATGCAGTGGATCTGATTGCAGAAGGCAAATTCGATCGTATGGTTGCATGGAGTGATCGCGGT
GTGATTGATGTTCCGATTACCGATGCCATTGCCAAATATGCCTGCGTGGAACTGGATGGTGCAATGGTTAAAACC
GCCCGTGGTCTGGGCATTAGTCTGGGCGATTAA

Figure S10. Codon optimized sequences of ADP-PFK-As from cyanobacteria and α-proteobacteria for expression in *E. coli* (changes in red)

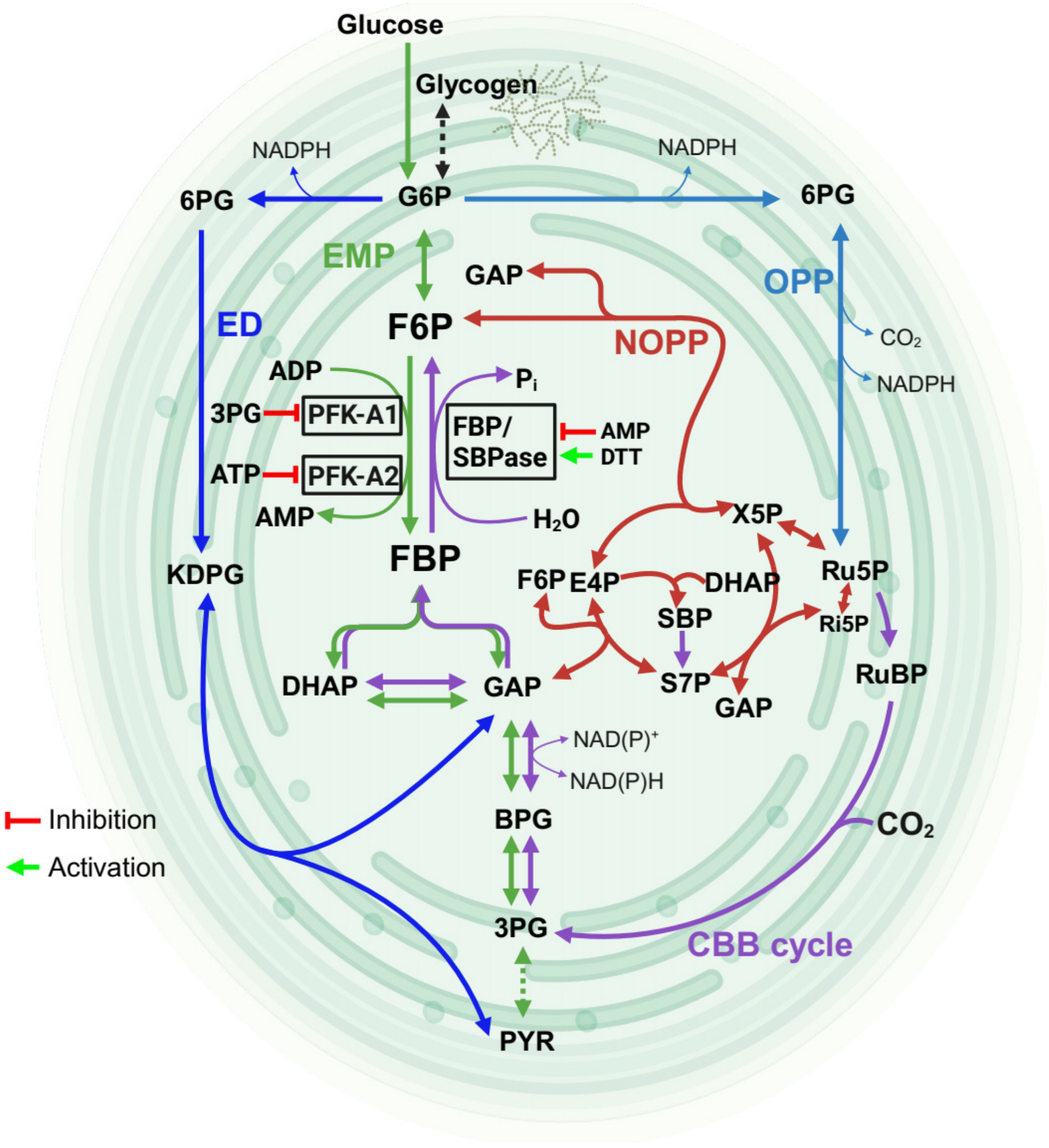

Fig. S11. Overview of pathways involved in CO_2 fixation and carbohydrate metabolism in *Synechocystis* sp. PCC6803, with a focus on the regulation at the level of fructose 6-phosphate and fructose 1,6-bisphosphate conversion. The key enzymes of the reversible EMP pathway, phosphofructokinase (PFK-A1 and PFK-A2) and the bifunctional fructose-1,6-bisphosphatase/sedoheptulose-1,7-bisphosphatase (FBP/SBPase) and their regulation are shown. Pathways are differentiated by arrow color: The EMP pathway is shown in green, the OPP pathway in cyan, the ED pathway in blue, the CBB cycle in purple, and the NOPP pathway in red. The PK pathway was omitted for clarity. Abbreviations: EMP, Embden–Meyerhof–Parnas pathway; OPP, oxidative pentose phosphate pathway; NOPP, non-oxidative pentose phosphate pathway; CBB, Calvin–Benson–Bassham cycle; G6P, glucose 6-phosphate; F6P, fructose 6-phosphate; FBP, fructose 1,6-bisphosphate; DHAP, dihydroxyacetone phosphate; GAP, glyceraldehyde 3-phosphate; BPG, 1,3-bisphosphoglycerate; 3PG, 3-phosphoglycerate; PYR, pyruvate; 6PG, 6-phosphogluconate; Ru5P, ribulose 5-phosphate; X5P, xylulose 5-phosphate; Ri5P, ribose 5-phosphate; RuBP, ribulose 1,5-bisphophate; E4P, erythrose 4-phosphate; S7P, sedoheptulose 7-phosphate; SBP, sedoheptulose 1,7-bisphosphate; [H], high concentration of reducing equivalents. The figure was created using BioRender.com.

Table S1. MS parameters of native MS analysis of PFK-A1 and PFK-A2.

Parameter	PFK-A1	PFK-A2
m/z range	1,000-15,000	2,000-10,000
Capillary temperature [°C]	150	150
Microscan count	10	1
FT resolution	17,500	8,750
AGC target	$3 \cdot 10^6$	$3 \cdot 10^6$
HCD	0	200
UHV sensor [mbar]	$5.8 \cdot 10^{-10}$	$5.6 \cdot 10^{-10}$

Table S2. Non-standard UniDec software settings used in the native MS data analysis.

Parameter	PFK-A1	PFK-A2
Data Processing:		
m/z range	4,000-9,000	3,000-8,000
Background Subtraction	Yes	Yes
UniDec Parameters:		
Mass Range [Da]	20,000-400,000	20,000-500,000
Sample Mass Every [Da]	1	1
Beta	2	0
Point Smooth Width	15	1
Peak Selection:		
Peak Detection Threshold	0.01	0.01
Peak Normalization	Total	Total
Additional Filtering:		
Filter Peak Scores [% DScore]	30	40

Table S3. Intracellular concentrations for metabolites that affect PFK activity in both photoautotrophic and photomixotrophic growth contexts. Intracellular concentrations were calculated from Takahashi et al. 2008 (44), using a conversion factor of 0.249 ml/g FW. *The F6P concentration is calculated from the G6P concentration assuming that the phosphoglucoseisomerase reaction is in chemical equilibrium with a Keq of 0.314.

Metabolite	Photoautotrophic (mM)	Photomixotrophic (mM)
3 PG	5.39	0.41
ATP	2.33	2.28
ADP	2.18	3.12
AMP	0.87	1.54
G6P	0.49	9.02
F6P*	0.15	2.83
